# Early Life Stress Affects Human Decision Making by Increasing Expectations of Volatility

**DOI:** 10.1111/desc.70238

**Published:** 2026-06-17

**Authors:** Karen E. Smith, Payam Piray, Alyssa Teal, Selin Gok, Seth D. Pollak

**Affiliations:** ^1^ Department of Psychology Rutgers University Newark New Jersey USA; ^2^ Department of Psychology University of Southern California Los Angeles California USA; ^3^ Department of Psychology & Waisman Center University of Wisconsin–Madison Madison Wisconsin USA

**Keywords:** adversity, computational modeling, decision making, development, early life stress, learning

## Abstract

People learn most effectively when they can flexibly modify strategies to accommodate environmental changes. Here, we explore how chronic early life stress influences the ways individuals weight and prioritize new information when making decisions. To do so, we examined the choices of 11–16‐year‐old children in a reward learning task. Children varied on their level of stress exposure. We used a Bayesian computational learning model to assess whether differences in children's decision making were driven by difference in their expectations about the task environment. Children with high stress exposure switched their responses rather than maintaining choices that had previously been effective for them when making decisions about rewards. This switching was driven by children with high stress exposure expecting environments to be more volatile. These findings offer insight into how early life experiences can shape the subsequent parameters of human learning.

**Summary:**

- How experience shapes an individual's ability to flexibly adjust learning strategies when making decisions is not well understood.
- Using behavioral tasks combined with a novel computational model, we find exposure to high stress environments biases children towards learning strategies optimal to unpredictable environments.
- Stress in childhood shapes the development of learning parameters in ways that have implications for decision making.

## 1 Introduction

To effectively navigate their environments, children need to be able to adjust their learning strategies dependent on changing environmental contexts. Rapidly adjusting strategies in response to changes in the learning environment allows for more optimal decision making (Daw and Tobler 2013; Padoa‐Schioppa and Assad 2006). When an environment is relatively stable and predictable, individuals can achieve optimal outcomes by adjusting their behavior to new information more slowly and deliberately—for example, a child whose route to walk to school every day is consistently 20 min with reliable crossing guards at the busy intersections will not change the route if one day the crossing guard is not there and they do not make it to school on time. This is because in a highly stable context, changes may reflect noise or less meaningful information over the long‐term. However when an environment is volatile, unpredictable, and frequently changing, prioritizing recent outcomes and new information represents the more optimal strategy as each change is more likely to be meaningful (Behrens et al. 2007; Massi et al. 2018; Weiss et al. 2021; Xu et al. 2023)—for example, sidewalk closures and unreliable crossing guards may result in a child changing their route to walk to school more frequently. Adults and children generally adjust their behaviors to their learning environment, and this ability increases with age (Browning et al. 2015; Habicht et al. 2022; Iglesias et al. 2013; Nussenbaum et al. 2022) However, it is less clear how early experiences shape the development of this ability. The present study tests the hypothesis that features of children's early environments influence the way they interpret and prioritize new information when making future decisions.

One feature that might influence decision‐making during development is exposure to stress. Exposure to extreme stressors and adversity, including things like maltreatment, living in poverty, and domestic violence, in childhood is associated with both disrupted behavioral performance and altered neural activity during reward learning (Birn et al. 2017; Gonzalez et al. 2016; Morelli et al. 2021). Children exposed to high levels of stress early in life also have difficulty learning contingent relationships (Fareri and Tottenham 2016; Galván 2014; Harms et al. 2018). These disruptions, in turn, appear to contribute to a host of social and behavioral problems (Herzberg and Gunnar 2020; Palacios‐Barrios and Hanson 2019; Wismer‐Fries and Pollak 2017). One reason for why stress shapes learning is that extreme stressors are characterized by high levels of volatility and uncertainty (McEwen and Akil 2020; McEwen and McEwen 2017; Peters et al. 2017; Smith and Pollak 2021). Indeed, stress has been demonstrated to heighten expectations of uncertainty (de Berker et al. 2016) and increase exploitation and avoidance when making decisions (Lenow et al. 2017; Smith and Pollak 2022). Chronic exposure to highly unpredictable environments early in life could bias children expecting high levels of volatility in future learning environments.

Here, we examine whether chronic stress exposure biases children toward expectations of high environmental volatility when making decisions. To do so, we modeled trial‐by‐trial learning as children with varying levels of early life stress made decisions about whether to switch or stay with previously rewarded options in stable and unstable learning contexts. We expect that, in more volatile contexts, children will update action‐outcome relationships more quickly in response to new information (Behrens et al. 2007; Meder et al. 2017). Additionally, we expect that children with high levels of stress exposure will switch their choices frequently regardless of the actual stability of the cue‐reward pairings. If this increased switching is reflective of increased perceived volatility, we expect this increased switching to be driven by recent feedback. Alternatively, if it is reflective of overall impaired ability to accurately detect and respond to changes in the environment, we expect it to be random and independent of feedback.

## 2 Method

### 2.1 Participants

The sample included 89 11–16‐year‐olds (48 female; M = 13.42, SD = 1.44; Race: 68.5% White, 2.2% White Hispanic/Latino, 1.1% Asian, 15.7% Black or African American, 1.1% Hispanic/Indian, 1.1% Native American, 9.0% Multiracial) recruited from the Madison, Wisconsin metropolitan area. This sample excluded an additional 3 subjects who were dropped due to issues during data collection. Given the lack of prior effects to estimate power, sample size was based on prior research on similar topics (Hanson et al. 2017; Richter et al. 2019) and recommendations from power simulation studies for hierarchical linear models (Kerkhoff and Nussbeck 2019). Parents provided consent for their child to participate, and participants provided assent and were paid $20 or $25. This study was approved by the University of Wisconsin ‐ Madison Institutional Review Board.

### 2.2 Procedure

**FIGURE 1:**
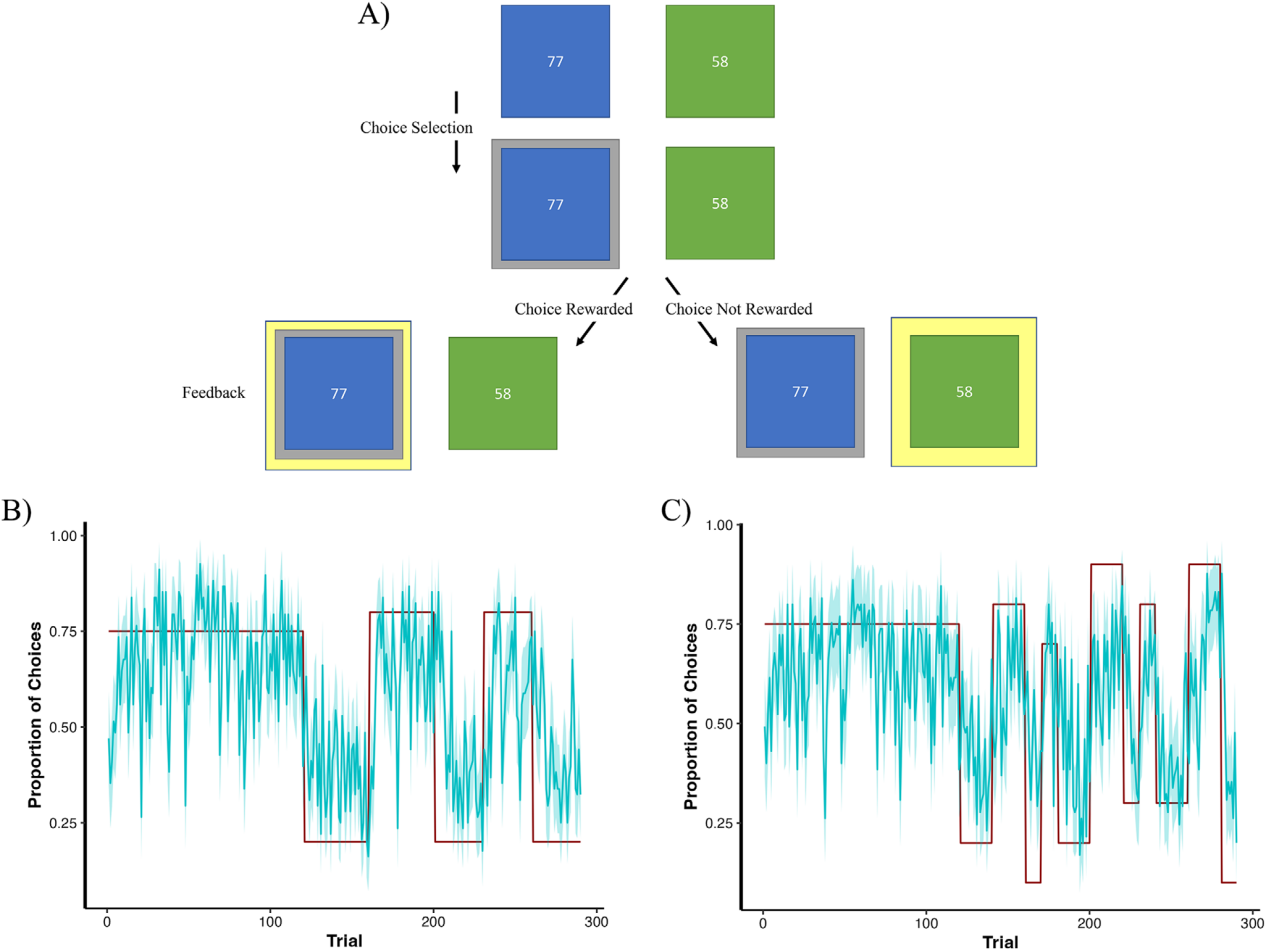
Probabilistic reward learning task. *Note*: (A) Participants were presented with two options containing potential reward values and instructed to select one. Numerical values in each box represent trial level reward magnitudes. Reward magnitudes varied trial by trial but were consistent within task versions. The option selected was highlighted in a gray box. After selection, participants then received feedback about which option was rewarded. The rewarded option was highlighted in yellow. Cumulative reward was indicated by a gray bar presented at the bottom of the screen that got longer when the selected choice was rewarded and stayed stationary when it was not. Participants completed a stable block (75% probability of reward of one option; e.g., blue) and either a somewhat volatile (switched from 80% to 20% every 30 or 40 trials) or volatile (alternated between 10%, 20%, 30%, 70%, 80%, and 90% every 10 to 20 trials) block. (B) Reward probabilities (red) and choice behaviors (blue) for the task version with a stable and somewhat volatile block and (C) stable and volatile block.

Participants completed a probabilistic reward learning task designed to assess their performance as levels of environmental volatility were manipulated. On each trial, participants chose between two colored boxes, each containing a different reward value (Figure 1). After making a choice, participants received feedback about whether the box they chose was the one that would be rewarded. If the box they chose was rewarded, participants received the reward value displayed in the box in the form of accumulated points. If the box they chose was not rewarded, they received nothing. Participants were told the number of points earned would determine their overall reward at the end of the study (though all participants received the same reward and were fully debriefed at the conclusion of the experiment). Consistent with similar task paradigms in this area (Behrens et al. 2007), total points earned were tracked using a gray bar at the bottom of the screen to maintain engagement with the task. The main feature of the task is that we manipulated the probability of which colored box was rewarded. Participants were told that each colored box would not be rewarded equally and that they can use information from previous trials to guide their choices; they were not given any other information about the underlying reward structure, nor were they told the reward structures would change over time. The probability a box was rewarded was either stable (75% probability of reward), somewhat volatile (switched from 80% to 20% every 30 or 40 trials), or volatile (alternated between 10%, 20%, 30%, 70%, 80%, and 90% every 10 to 20 trials). Probability of reward across the two boxes were reciprocal (e.g., if one box was rewarded at a probability of 75% the other box was rewarded at a probability of 25%). Participants completed two versions of the task during separate visits to the lab a year a part. At the first lab visit, participants completed a stable block of 120 trials and a somewhat volatile block of 170 trials. At the second lab visit, participants completed a stable block of 120 trials and a volatile block of 170 trials. Block order within each visit (e.g., stable first or second) was counterbalanced across participants. Of the 89 subjects, 44 completed both versions of the task, 24 completed only the somewhat volatile task version, and 21 completed only the volatile task version. This was due to the fact that it was not possible to recruit all participants who participated in the initial task version back to the laboratory. To keep group sizes relatively consistent, we opted to recruit an additional set of participants who had not participated in the first task to participate in the second. All reported analyses were also run including block order, block type (stable or volatile), task version (somewhat volatile or volatile), and whether participants completed both versions, along with higher order interactions of all covariates with lifetime stress exposure.

### 2.3 Stress Exposure

We assessed children's lifetime stress exposure using the Youth Life Stress Interview (Rudolph et al. 2000). The Youth Life Stress Interview is a widely used semi‐structured interview measure of cumulative child stress. Children and parents are asked to independently report on children's experiences of stress including both events (e.g., experiences with death in the family, detention/suspension, peer conflict) as well as children's perceptions of and reactions to those experiences. General and specific probes are used to assess exposure to the different events and circumstances. Semi‐structured follow‐up questions are then asked to assess the event's context. Interviews were conducted by trained clinical staff. A separate group of trained raters with no knowledge of the child's subjective state scored the interview data on a 10‐point scale reflecting cumulative life stress and provided a consensual rating that reflected the overall level of stress. This rating incorporates a detailed consideration of the context of events and impact on the individual child's life rather than reflecting simply the number of stressors. Specific scoring details can be found in (Hanson et al. 2015). One subject was missing interview scores and dropped from analyses.

### 2.4 Computational Model

We evaluated participants’ choice data in the probabilistic reward learning task using a hidden Markov model (HMM), used in Bayesian machine learning to model sequential binary data (Bishop 2006). This approach represents a principled approach to modeling volatility (Bishop 2006; Ghahramani 2001). The HMM framework is particularly appropriate for scenarios involving binary hidden states, and has been used to fit human choice data in reversal learning tasks (Hampton et al. 2006; Schlagenhauf et al. 2014). In our task, the binary hidden state represents which of the two colored boxes currently has the higher reward probability; this state switches over time in volatile environments but remains stable in non‐volatile ones. For the present study, the key advantage of using an HMM is its robustness in parameter estimation and interpretability (as shown by Fang and Piray 2025; Piray and Daw 2020), making it especially suitable for analyzing individual differences. In our recent work, we have also shown that the HMM performs well in capturing human learning under uncertainty, both in terms of capturing the average effects, and in revealing meaningful variation across individuals (Fang and Piray 2025).

Particularly relevant to the current study, the HMM approach extends the power of the models frequently applied to learning by explicitly estimates two key aspects of learning environments that contribute to uncertainty (Piray and Daw 2021). The first is volatility, which refers to how quickly the underlying hidden state (i.e., which colored box is currently better) changes. The second is noise, which refers to the degree of observation or measurement noise in each outcome around the value of its cause. By allowing these parameters to vary across individuals, we can quantify each participant's implicit expectations about both noise and environmental volatility, a measure particularly relevant for understanding how stress might alter perception of environmental stability. In this modeling approach these two parameters are allowed to vary across individuals, resulting in indices of individual level expectations of environmental volatility and noise. This is in contrast to previous models which treat one or both as fixed (Behrens et al. 2007; Mathys et al. 2011; Piray and Daw 2020), but similar to approaches used previously for studying psychopathology (Browning et al. 2015). This model takes advantage of the fact that in environments with high levels of volatility, individuals not only increase the rate at which they adjust behaviors to new information (resulting in increased switching in behavioral choices) but exhibit highly autocorrelated choices. In contrast, in environments with high levels of noise, individuals will demonstrate increased switching behaviors, but they will not necessarily depend on feedback and will not be highly autocorrelated.

We consider modeling outcomes for only one of the colored boxes as outcomes for the other box are simply the inverse probability of the modeled box. In this model, outcome on trial *t*, *o_t_*, is a binary (0 or 1) variable that depends on a hidden binary variable, *x_t_*. The hidden variable has a Markov structure depending on the volatility parameter *v*:

$$\;x_{t}=\left\{\begin{array}{c} x_{t-1},\;\mathrm{with}\;\mathrm{probability}\;1-v\\ 1-x_{t-1},\;\mathrm{with}\;\mathrm{probability}\;v \end{array}\right.$$

Thus, the volatility parameter encodes the probability that the hidden variable switches. If *v*  =  0, the hidden variable on the current trial will certainly follow that on the previous trial. If *v*  =  0.5, the hidden variable will completely detach from its value on the previous trial. We always assume that *v* is between 0 and 0.5.

Outcomes are then generated based on the hidden variable and the noise parameter, *s*.

$$\;o_{t}=\left\{\begin{array}{c} x_{t},\;\mathrm{with}\;\mathrm{probability}\;1-s\\ 1-x_{t},\;\mathrm{with}\;\mathrm{probability}\;s \end{array}\right.$$

Thus, *s* encodes how noisy outcomes follow the hidden variable. If *s*  =  0, outcomes will certainly follow the hidden variable. The maximum noise occurs when *s*  =  0.5, in which outcomes will completely detach from the hidden variable. We always assume that *s* is between 0 and 0.5.

We modeled two volatility parameters for each participant, *v_s_* and *v_v_*, which respectively encode the volatility parameter for the stable and volatile block. We considered two different variants of this model: in one model both *v_s_* and *v_v_* are fit independently between in the range of 0 to 0.5, while in the other model, volatility in the stable block were constrained to be smaller than volatility for the volatility block, that is, *v_s_* < *v_v_*. While these models align with the structure of the task, they represent a simplified descriptive model from the participants' point of view—abstracting away the trial‐by‐trial dynamics wherein participants need to estimate the true, fixed blockwise parameters based on their noisy individual observations within each block. But for the purpose of the current study, this assumption yields a tractable algorithm that is robust and leads to stable model fitting and parameter recovery. If participants are tracking the underlying structure of the task blocks (stable as compared to volatile), the constrained model should be a better fit for participants’ behaviors. This expectation was confirmed by Bayesian model comparison techniques, which indicated the constrained model explained 68.9% of participants’ data as compared to the unconstrained model which explained 31.1% of participants’ data. To assess whether the model that best fit the data differed dependent on stress exposure, participants were split into high and low stress groups based on a median split. The constrained model best fit both high and low stress groups. To further confirm the constrained model systematically reproduced patterns of choice behavior similar to the participants, we simulated data using each participant's best fit parameters and found high correlations between simulated and actual data (*r*s > 0.59, *p*s < 0.001).

Inference about *x_t_* based on a series of outcomes is tractable for this generative process. It is straightforward to see that the posterior probability for the hidden variable has a Bernoulli distribution $p\;\left(\;x_{t}=1|o_{1},\text{…},o_{t}\right)=r_{t}$, and it is possible to obtain *r_t_* based on the posterior on the previous trial. In particular, the probability based on observations until the previous time‐point, $p\;\left(\;x_{t}=1|o_{1},\text{…},o_{t-1}\right)={\hat{r}}_{t}$ is given by

$$\;{\hat{r}}_{t}=r_{t-1}\;\left(1-v\right)+\left(1-r_{t-1}\right)v$$

Next, *r_t_* is obtained based on ${\hat{r}}_{t}$ and the outcome observed on the current trial, *o_t_*

$$\;r_{t}=\left\{\begin{array}{c} \left(1-s\right){\hat{r}}_{t}/\left(1-s\right){\hat{r}}_{t}+s\left(1-{\hat{r}}_{t}\right),\;\mathrm{if}\;o_{t}=1\\ s{\hat{r}}_{t}/s{\hat{r}}_{t}+\left(1-s\right)\;\left(1-{\hat{r}}_{t}\right),\;\;\mathrm{if}\;o_{t}=0 \end{array}\right.$$

We then combine the predicted probabilities from the HMM with a softmax choice model. In particular, the predicted value of the modeled box, *V_M_*, on trial *t* is given by its probability times its magnitude, *r*_*t* − 1_ *M_t_*, where *M_t_* is the magnitude of the box on the current trial. Similarly, the predicted value for the other box, *V_O_*, is given by (1 − *r*_*t* − 1_)*O_t_*, where *O* is the reward magnitude of the box on the current trial. The probability of choosing the modeled box is then given by

$$p\;\left(\;c_{t}=modeled\;box\right)=\frac{1}{1+\exp\left(-\beta \left(V_{M}-V_{O}\right)-\phi S\right)}$$

where β is the decision coefficient parameter, ϕ is the perseveration parameter, and *S* is the repeat function which is 1 if the choice on the previous trial was blue, otherwise it is − 1. Models were fit separately for each participant.

For each participant, we fit five parameters (*v_s_*, *v_v_*, *s*, β and ϕ) to their choice data. We employed a standard gradient‐based optimization method for model fitting (Piray et al. 2019). The prior mean and variance for all parameters were set to 0 and 1, respectively. The parameter fitting was performed in the infinite real‐space and later transformed to obtain the actual parameters used by the models. Suitable sigmoid or exponential transform functions were applied for this purpose. See Supporting Information for more information on model fitting, model comparison, and model recovery processes.

## 3 Results

**FIGURE 2:**
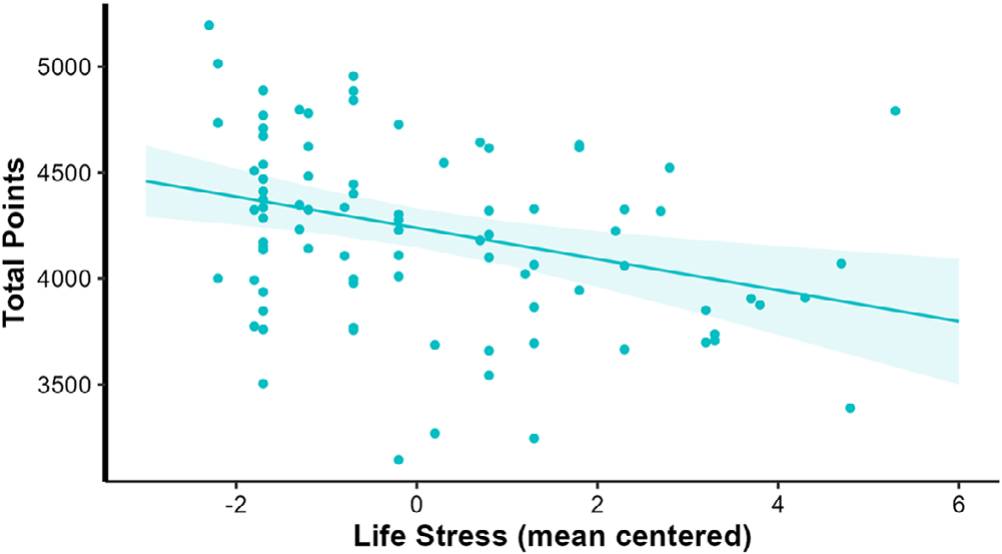
Relationship between lifetime stress exposure and average points won in the task. *Note*: Higher levels of lifetime stress exposure were associated with decreased performance. Simulating random responses for 1000 participants indicated random performance would result in a mean total points value of 3560.25. Shading represents the confidence band.

We took a three‐step approach to determining whether lifetime stress exposure increased children's expectations of volatility. First, we examined whether children with higher levels of stress exposure demonstrate poorer performance in the reward learning task, which would be consistent with the extant literature. Next, we used a theory neutral approach to examine what is driving these differences in performance—in particular, whether children with higher lifetime stress exposure switch their behaviors more frequently, indicative of increased uncertainty. Last, we employed the computational modeling approach outlined above to test whether this switching behavior was driven by increased expectations of environmental volatility. Unless otherwise specified, we used a multilevel modeling framework for all reported analyses, with continuous predictors mean centered to increase interpretability of the model intercept and reduce issues associated with collinearity (Raudenbush and Bryk 2002). Models were run using the lmer package in R v4.0.5 and included a random intercept for volatility task nested within participant and lifetime stress exposure as a fixed effect (see Supporting Information for full model details and equations). Replicating prior research, children with greater lifetime stress exposure demonstrated poorer performance on the reward learning task than their peers (*β* = ‐73.50, *SE* = 23.97, *p* = 0.002; Figure 2). Including volatility level, task version, and whether participants completed both task versions (somewhat volatile/volatile) along with all interactions did not change these effects. Children performed worse in the volatile blocks as compared to the stable (*β* = −3.89, *SE* = 0.39, *p* < 0.001) and children who completed both task versions performed better in the volatile task (*β* = 2.47, *SE* = 0.96, *p* = 0.01; Figure S1), potentially suggestive of some learning. However, there were no significant interactions between volatility level, task version, and completing both task versions with lifetime stressor exposure *(ps* > 0.10) suggesting that effects of stress exposure were consistent across volatility levels and independent of learning.

To examine whether the effect of lifetime stress exposure on task performance was driven by children's switching behaviors, we employed a relatively theory neutral approach and modeled participants’ trial‐by‐trial behavioral choices to stay with or switch to a new option dependent on reward history. To do so, we analyzed the “stay” behavior (i.e., remaining with the same choice) using a logistic regression analysis including a reward regressor, encoding whether the choice on the previous trial was rewarded, and a stability regressor, encoding whether trials are stable. This approach resulted in three parameters: (1) an intercept, which encodes the tendency to stay with the previous choice regardless of the reward history; (2) reward regressor effect, which encodes the effects of reward of previous trial on stay behavior; (3) stability regressor effect, which encodes the effects of trial stability on stay behavior. Individual level parameters were generated. The effects of lifetime stress on parameters were then analyzed using the multilevel modeling framework described above. Most children were sensitive to reward probabilities and stayed with their prior choice when the probability of those choices being rewarded was high (reward regressor: *β* = 0.01, *SE* = 0.001, *p* < 0.001; Figure 1), suggestive of successful learning. Additionally, children were more likely to stay with their prior choice following stable trials (stability regressor: *β* = 0.07, *SE* = 0.02, *p* = 0.003; Figure 1), indicative of adaptation to the underlying task structure. However, individuals who experienced high lifetime childhood stress were more likely to switch their choices (i.e., less likely to stay with the prior choice as indexed by the intercept; *β* = −0.05, *SE* = 0.03, *p* = 0.05), less sensitive to reward probabilities (reward regressor: *β* = −0.001, *SE* = 0.0004, *p* = 0.031), but showed no difference in likelihood of staying with their prior choice following stable trials (stability regressor: *β* = −0.004, *SE* = 0.01, *p* = 0.72; Figure S2). Including volatility level, task version, and whether participants completed both task versions (somewhat volatile/volatile) along with all interactions resulted in the effects of lifetime stress on switching (*β* = −0.05, *SE* = 0.03, *p* = 0.08) becoming trending and reward no longer significant (*β* = ‐0.001, *SE* = 0.0005, *p* = 0.10). However, the effects on switching and stability sensitivity were significant after inclusion of all other covariates (*p*s < 0.01). There were no effects of volatility level, task version, and whether participants completed both task versions or interactions with lifetime stress exposure on switching behaviors or sensitivity to reward (*p*s > 0.10). There was an interaction between lifetime stress exposure and task version on the stability regressor (*β* = ‐0.06, *SE* = 0.03, *p* = 0.04) such that lifetime stress exposure was positively with staying with the prior choice following stable trials in the somewhat volatile task version (*β* = 0.03, *SE* = 0.02, *p* = 0.18) and negatively in the volatile task version (*β* = −0.03, *SE* = 0.02, *p* = 0.15; Figure S1). However, neither slope was significantly different from zero. There were no other effects volatility level, task version, and whether participants completed both task versions on the stability regressor (*p*s > 0.09).

We then tested whether this increased switching behavior was indicative of increased prior expectations of environmental volatility. To do so we used a computational model that estimates expectations of environmental volatility. The model assumes that the agent must draw inferences about a hidden variable (e.g., the blue box being the correct choice) from uncertain binary outcomes (e.g., whether the blue box was rewarded or not) that are corrupted by two distinct sources of uncertainty: volatility and outcome noise. Inferences about these two sources of uncertainty will be reflected in the agent's behavior. This modeling approach therefore extends the theory‐neutral analysis presented above in two important ways: (1) by quantifying the effects of these two uncertainty parameters on learning; and (2) by capturing the long‐term effects of reward on choice (rather than only focusing on the previous trial). We fit parameters of this model to each subject's choice dataset independently using Bayesian model fitting methods that we and others have used in the past (Piray et al. 2019).

**FIGURE 3:**
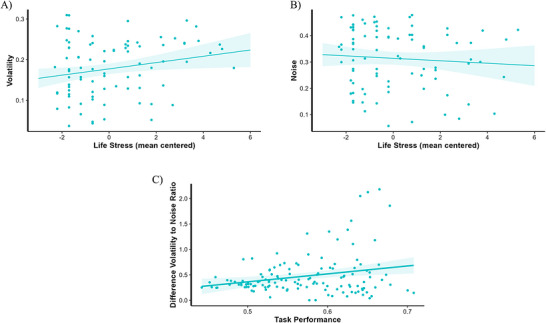
Relationship between lifetime stress exposure and the volatility and noise parameters. *Note*: Higher levels of lifetime stress exposure were associated with increased expected volatility (A) but not expected noise (B). Adaptive behavior requires a higher volatility‐to‐noise ratio in the volatile block compared to the stable block (Piray and Daw 2024). Thus, we analyzed this ratio for all participants by fitting the model and calculating the difference in volatility‐to‐noise ratios between blocks (C). This model‐derived metric correlated significantly with task performance, measured by the average number of rewarded choices (*r* = 0.25, *p* = 0.004). This relation did not differ based on lifetime stress exposure (β = 0.12, SE = 0.29, *p* = 0.67). Shading represents the confidence band.

Consistent with our hypothesis, children with higher lifetime stress demonstrated increased expectations of volatility (*β* = 0.01, *SE* = 0.003, *p* = 0.03; Figure 3A). There was no effect of lifetime stress on the noise parameter (*β* = −0.005, *SE* = 0.01, *p* = 0.46; Figure 3B), suggesting increased switching is due to increased shifts in expectations of volatility rather than noise. Including volatility level in the model suggested children did adjust their behaviors to the volatility level, showing higher expectations of volatility in the volatile blocks (*β* = 0.12, *SE* = 0.005, *p* < 0.001) particularly for the volatile version of the task (*β* = 0.04, *SE* = 0.01, *p* < 0.001). There was also an interaction between lifetime stress and volatility level, suggesting the effects of lifetime stress on the volatility parameter were most pronounced at increased levels of volatility but only in the somewhat volatile task version (*β* = −0.01, *SE* = 0.005, *p* = 0.02; Figure S1). Children who completed both task versions did show reduced expectations of volatility (*β* = −0.03, *SE* = 0.01, *p* = 0.04) but did not demonstrate differences in expectations of volatility dependent on task version (*β* = −0.02, *SE* = 0.02, *p* = 0.38) or lifetime stress exposures (*β* = −0.02, *SE* = 0.01, *p* = 0.19). There were no effects of block, task version, and completion of both version or interaction with lifetime stress exposure on the noise parameter (*p*s > 0.10).

Including age, gender, general cognitive ability (using the digit‐span test from the Wechsler Intelligence Scale for Children; WISC‐V), depression (assessed with the Child Depression Inventory; Kovacs 1985), and anxiety (assessed with either the Revised Children's Manifest Anxiety Scale; *n* = 70; Reynolds and Richmond 1985; or Multidimensional Anxiety Scale for Children; *n* = 17; March et al. 1997) in the models did not change the effects of lifetime stress exposure. Analyses for the other model parameters not directly related to the a priori hypotheses are reported in the Supporting Information. All model outputs and full tables can be found on OSF (https://osf.io/34uhp).

## 4 Discussion

Adaptive learning requires balancing decision strategies that can flexibly accommodate a range of changing environmental contexts. Here, we find that children's early environments influence how they weight and prioritize new information when making decisions. Individuals with high levels of childhood stress exposure—contexts usually involving uncertainty and unpredictability—expect environments to be more volatile. This expectation is reflected in their choice of decision‐making strategies; children with higher lifetime stress exposure switch responses at a higher frequency rather than maintaining behavioral choices that had demonstrated prior efficacy. By understanding the assumptions individuals bring with them when confronting new learning situations, we can gain insight into the nature of decisions and behavioral choices that may appear maladaptive to an observer. The present data identify a potential explanation of the frequently documented association between childhood stress exposure and poor reward learning as well as offer insight into how early experiences can shape subsequent parameters of human learning.

The association between childhood stress and problems with reward learning is robust and well‐replicated (Birn et al. 2017). This deficit is implicated in a range of poor social, educational, and health related outcomes (VanTieghem and Tottenham 2018). Therefore, one way to interpret the present data is that early life stress biases children toward assumptions of high environmental volatility, impairing their ability to make optimal decisions given the features and structures of a given environment. A second, and not mutually exclusive, interpretation is that these data reflect the ways in which humans adaptively configure learning to adverse environments. In a context marked by high levels of instability, calibrating decisions based upon volatility likely allows children to maximize positive outcomes and avoid potential threats. This is because when action‐outcome relationships are changing rapidly, switching behaviors more often to account for these changes results in more efficient learning (Farashahi et al. 2017; McGuire et al. 2014).

However, we did not find that increased expectations of volatility translated to better performance in the volatile blocks of the tasks. This may result from the fact that adaptive learning also requires adjusting strategies to accommodate the features of not one, but many different environments; early stress exposure may undermine this cognitive flexibility (Harms et al. 2018). When an environment is relatively stable and predictable, efficient learners decrease the speed at which they update action outcome associations, adjusting their behavior to new information more slowly and deliberately (Farashahi et al. 2017; Lee et al. 2020). This is because in a highly stable context, changes may reflect noise or less meaningful information over the long‐term. However, when an environment is volatile, unpredictable, and frequently changing, prioritizing recent outcomes and new information represents the more optimal strategy as each change is more likely to be meaningful. We did find that children adjusted to the underlying structure of the environment, demonstrating increased expectations of volatility in the more volatile task version and volatile blocks. But these effects were not modified by lifetime stress exposure. Together these findings suggest lifetime stress exposure shifts children toward expecting environments to be more volatile and this does not differ depending on the underlying environmental statistics. This could be representative of decreased flexibility in effectively adjusting their expectations to underlying environmental probabilities. This lack of flexibility is in line with research finding that children in highly unpredictable environments demonstrate decreased inhibitory control (Mittal et al. 2015) and more stimulus‐driven responses (Xu et al. 2023), but also tend to demonstrate better performance on executive functioning tasks that require rapid switching (Fields et al. 2021). This inflexible decision making could reflect difficulties in either *learning* or *using* the value of information from the environment to guide future choices (Smith and Pollak 2022). However, future research replicating the current findings and assessing learning and use separately is necessary to determine the specific mechanisms through which early stress shapes decision making.

The current research focused on how individual differences in children's lifetime stress relates to estimates of volatility, while holding stochasticity (i.e., outcome noise) in the learning environment constant. While this approach aligns with prior work (Behrens et al. 2007; Deserno et al. 2020; Diaconescu et al. 2020; Iglesias et al. 2013; Mathys et al. 2011; Nassar et al. 2012), recent research emphasizes the importance of dissociating volatility from stochasticity when both are unknown and potentially changing (Fang and Piray 2025; Piray and Daw 2021, 2024). We found minimal evidence for an effect of lifetime stress exposure on expectations of noise. However, to better determine how early stress shifts expectations of volatility and noise across different environments, research manipulating both volatility and stochasticity simultaneously is necessary. Additionally, our modeling approach does not explicitly separate the detection of environmental volatility from the tracking of reward contingencies within volatile contexts. A more sophisticated approach would involve hierarchical two‐layer models, in which one layer determines whether the environment is stable or volatile, and a second layer, conditional on volatility, monitors state changes in reward probabilities. Such architectures combine a lower‐level model (e.g., HMM or Kalman filter) with a higher‐level component that tracks volatility using sequential sampling methods such as particle filters (Fang and Piray 2025; Piray and Daw 2021, 2024). Although promising, these models face a critical challenge in parameter estimation: the likelihood functions produced by particle filter–based components are nondifferentiable, which currently limits their application to the study of individual differences. While hierarchical models may ultimately provide deeper insights into why some participants are less responsive to volatility, fitting and validating them remains an important direction for future research. Finally, some participants completed both versions of the tasks while other completed only one, introducing the potential for practice effects. However, we observed minimal evidence of practice effects over the one‐year time period and completion of both task versions did not modify the effects of lifetime stress exposure. Future research can collect both longitudinal and cross‐sectional samples to better understand how these abilities change over time and assess their generalizability across populations.

Experiences of stress are neither unidimensional nor captured simply by event exposures. Whether an individual comes to perceive a situation as stressful depends upon multiple factors, including but not limited to unpredictability, social support, and other factors (Sapolsky 2015; Smith and Pollak 2026). The Youth Life Stress Interview used in the current study captures aspects of some of these factors. A useful step in future research will be to better understand the developmental factors that influence how children perceive their environments. A second fruitful future direction will be to disentangle how childhood stress and unpredictability influence different components of decision making. Task demands can influence the way expectations of volatility are manifested. For example, in environments involving high levels of risk, children may tend toward more habitual behaviors, particularly if they have experienced high levels of unpredictability (Lin et al. 2022; Smith et al. 2025; Xu et al. 2023). Therefore, tasks framed around contexts of both risk and reward in larger and more diverse samples can assist in understanding what motivates different types of behaviors. Finally, our sample also spanned developmental periods of pre‐adolescence into adolescence, periods that represent rapid change and reorganization across multiple systems involved in stress responding and valuation (Galván 2014; Marceau et al. 2023). Future research examining how effects during this period compare to early and mid‐childhood, along with how puberty may influence the effects, can better inform our understanding of how stress shapes learning and decision making processes.

The current experiment expands our understanding of how early life experiences can shape the parameters of subsequent human learning. Stressful environments may help children prioritize some types of information in highly unstable environments but may also decrease their flexibility to adjust as their contexts change. This research points to altered learning processes being one mechanism through which early life stress may shape later developmental outcomes. Individuals who have difficulty flexibly responding to their circumstances may miss opportunities for deeper learning and may therefore navigate less adaptively through their social worlds. These insights into the development of decision‐making further reveal the myriad ways in which early experiences exert broad effects on the way humans learn from and adapt to their changing circumstances.

## Author Contributions

**Karen E. Smith**: formal analysis, writing – review and editing, writing – original draft, data curation, visualization, methodology. **Alyssa Teal**: writing – review and editing, project administration, investigation, methodology. **Selin Gok**: investigation, writing – review and editing. **Payam Piray**: writing – original draft, writing – review and editing, formal analysis, data curation, methodology, funding acquisition, visualization. **Seth D. Pollak**: conceptualization, funding acquisition, writing – review and editing, methodology, project administration, supervision, resources.

## Funding

This research was supported by the National Institute of Mental Health R01MH61285 (SDP), T32MH018931‐30 (KES), R21MH134217 (PP)] and by a core grant to the Waisman Center from the National Institute of Child Health and Human Development [U54 HD090256].

## Conflicts of Interest

The authors declare no conflicts of interest.

## Supporting information




**Supporting File 1**: desc70238‐supp‐0001‐SuppMat.docx

## Data Availability

Associated data and analysis scripts are available on OSF (https://osf.io/34uhp).
