# Supplementary material for: Early Life Stress Affects Human Decision Making by Increasing Expectations of Volatility
Source: Dev Sci. 2026 Jun 17;29(4):e70238. doi: 10.1111/desc.70238 (PMC13275639; doi:10.1111/desc.70238)
Supplement: Supplementary file 1 — Supporting File 1: desc70238‐supp‐0001‐SuppMat.docx [file DESC-29-e70238-s001.docx]

Supporting Information

Additional Methods

**Model Fitting and Model Comparison**

We fit parameters independently for each participant by maximizing the log posterior, i.e., the log likelihood plus the log prior assuming a prior mean and variance of 0 and 1 for all parameters, respectively. Model parameters were fit in an infinite real-space, and they were transformed to obtain actual model parameters using appropriate functions (i.e., sigmoid for parameters bounded between 0 and 1 and exponential for positive parameters.) For model comparison, we employed a random effects model selection approach (Piray et al., 2019; Rigoux et al., 2014). Prior to model fitting, we conducted a recovery analysis to test whether model parameters were recoverable. To do this, 100 artificial choice datasets were generated using a set of known parameters. For model fitting, parameters were treated as unknown and the same Bayesian model fitting method was used to find them only based on simulated datasets. This analysis confirmed that the model parameters were recoverable (Figure S3). Additionally, there was no tradeoff in recovery of the two volatility parameters (for the volatile and stable blocks), as they were both highly recoverable. Posterior predictive checks were conducted in which choices were simulated from the posterior predictive distribution. We then re-ran theory neutral analyses using the simulated choices. These analyses replicated those reported in the paper, with lifetime stress exposure being associated with increased switching (*β* = -.05, *SE* = .02, *p* = .03; Figure S4), suggesting the model adequately fit the data. In addition, to confirm computational parameters captured model free behavior, we ran correlations between all model parameters and theory neutral parameters. Correlations between parameters were in the expected direction (volatility and noise associated with reduced stay behavior; perseverance associated with increased stay behavior; Figure S5).

**Multi-level Model Equations**

To examine the effects of lifetime stress exposure on aggregate indicators of performance on the task (points won and choice behaviors) and expected volatility, we ran a series of three-level multi-level models with block nested within task version nested within participant. This structure accounts for the repeated measures nature of the data while also accounting for the fact some participants are missing data for each time point (Curran et al., 2010; Singer & Willett, 2003). Given our primary questions, were related to the effects of lifetime stress on behaviors, an initial model including only lifetime stress as a fixed subject level effect was run:

Level 1 (Block) Equation:

$$Y_{jki}=\pi_{0ki}+\varepsilon_{jki}$$

Level 2 (Task Version) Equation:

$$\pi_{0ki}=\gamma_{00i}+\zeta_{0ki}$$

Level 3 (Participant) Equation:

$$\gamma_{00i}=\delta_{000}+ \delta_{001}STRESS+V_{0i}$$

Composite Equation:

$$Y_{jki}=\delta_{000}+ \delta_{001}STRESS+\varepsilon_{jki}+ \zeta_{0ki}+V_{0i}$$

Where $Y_{ijk}$ represents the outcome for individual *i* during task version *k* during block *j*; $\pi_{0ki}$ represents the level 1 intercept; $\gamma_{00i}$ the level 2 intercept; $\delta_{000}$ the level 3 intercept, and $\delta_{001}$ the effect of lifetime stress exposure. $\varepsilon_{jki}, \zeta_{0ki}, \mathrm{and} V_{0i}$ represent the level 1, level 2, and level 3 residuals (random effects).

Then models were run also including fixed effects of task version, block, order of presentation, and whether participants completed both task versions to assess whether effects differ dependent on these factors:

Level 1 (Block) Equation:

$$Y_{jki}=\pi_{0ki}+\pi_{1ki}Block+\varepsilon_{jki}$$

Level 2 (Task Version) Equation:

$$\pi_{0ki}=\gamma_{00i}+ \gamma_{01i}TaskVersion+ \gamma_{02i}BlockOrder+ \gamma_{03i}BothTasks+\zeta_{0ki}$$

$$\pi_{1ki}=\gamma_{10i}+ \gamma_{11i}TaskVersion+ \gamma_{12i}BlockOrder + \gamma_{13i}BothTasks$$

Level 3 (Participant) Equation:

$$\gamma_{00i}=\delta_{000}+ \delta_{001}STRESS+V_{0i}$$

$$\gamma_{01i}=\delta_{010}+ \delta_{011}STRESS$$

$$\gamma_{02i}=\delta_{020}+ \delta_{021}STRESS$$

$$\gamma_{03i}=\delta_{030}+ \delta_{031}STRESS$$

$$\gamma_{10i}=\delta_{100}+ \delta_{101}STRESS$$

$$\gamma_{11i}=\delta_{110}+ \delta_{111}STRESS$$

$$\gamma_{12i}=\delta_{120}+ \delta_{121}STRESS$$

$$\gamma_{13i}=\delta_{130}+ \delta_{131}STRESS$$

Composite Equation:

$$Y_{jki}=\delta_{000}+ \delta_{001}STRESS+V_{0i}+ \delta_{010}TaskVersion+ \delta_{011}STRESSTaskVersion+ \delta_{020}BlockOrder+ \delta_{021}STRESSBlockOrder+ \delta_{030}BothTasks+ \delta_{031}STRESSBothTasks+\delta_{100}Block+ \delta_{101}STRESSBlock+ \delta_{110}TaskVersionBlock+ \delta_{111}STRESSTaskVersionBlock+ \delta_{120}BlockOrderBlock+ \delta_{121}STRESSBlockOrderBlock + \delta_{130}BothTasks+ \delta_{131}STRESSBothTasks+\varepsilon_{jki}+\zeta_{0ki}+V_{0i}$$

Analyses for the other model parameters and theory neutral model followed the same design, but did not include a fixed effect of block. For all models, categorical predictors were sum to zero effect coded to increase interpretability of any main effects. All models were run also including age in year, gender, general cognitive ability, depression, and anxiety as participant level fixed effects to assess whether effects hold while controlling for these factors. All continuous predictors were mean centered except for anxiety. Given different measures of anxiety were collected from different subsets of participants, values were standardized to increase comparability.

Additional Results

**Child Behavior**

On average for each task version, children won 4237.66 points (*SD* = 491.40), chose the option with a high probability of reward 62.74% of the time (*SD* = 12.03), and had their chosen option reward 57.19% of the time (*SD* = 7.64). Lifetime stressful event exposure was associated with how many points children won during the task (*β* = -73.50, *SE* = 23.97, *p* = .002), such that children with greater lifetime stress exposure won fewer points (Figure 2). There was also a trending effect of lifetime stress exposure on choice behaviors, such that children with higher lifetime stress more often chose the option with less probability of reward (*β* = -.01, *SE* = .005, p = .06; Figure S6) and on average had a smaller frequency of rewarded choices (*β* = -.01, *SE* = .003, *p* = .09; Figure S6). Including block, task version, block order, and whether participants completed both task versions did not change these effects with the exception the effects on choosing the option with less probability of reward (*p* = .12) and frequency of rewarded choices (*p* = .28) were no longer trending. Children did perform worse in the volatile blocks (average points won per trial: *β* = -3.89, *SE* = 0.39, *p* < 0.001; probability of choosing the option with a high probability of reward: *β* = -.06, *SE* = .01, *p* < .001; frequency of reward choices: *β* = -.03, *SE* = .01, *p* < 0.001), but this did not differ by stress exposure (*p*s > 0.05). For points won, children who completed both task versions performed better in the second task (*β* = 2.47, *SE* = 0.96, *p* = .01). There was a trending effect of completing both task version on frequency of rewarded choices (*β* = .02, *SE* = .01, *p* = .08) but not on probability of choosing the option with a high probability of reward (*β* = .02, *SE* = .02, *p* = .23). There were no interactions of lifetime stress exposure with volatility level, task version, or completing both version (*p*s > 0.10), except for choosing the option with a high probability of reward where lifetime stress exposure interacted with volatility level and task version (*β* = .02, *SE* = .01, *p* = .04) such that there was a negative relationship between lifetime stress exposure and choosing the high probability option only for the stable block in the volatile task version (*β* = -.02, *SE* = .01, *p* = .02)*.* Controlling for age, gender, cognitive ability, depression, and anxiety also did not change the effects of lifetime stress exposure with the exception the effect on frequency of rewarded choices was no longer trending (*p* = .23).

Analyses Other Model Parameters

There was no effect of lifetime stress exposure on the perseverance parameter (*β* = -.03, *SE* = .02, *p* = .11; Figure S7) but there was a negative effect of lifetime stress exposure on the decision coefficient parameter (*β* = -.003, *SE* = .001, *p* = .01; Figure S7). Controlling for task version, counterbalancing order, age, gender, general cognitive ability, depression, and anxiety resulted in the effects of lifetime stress on the perseverance (*p* = .06) and decision coefficient (*p* = .07) parameters becoming trending. Full parameter tables for all reported models can be found on OSF (<https://osf.io/34uhp>).

**References**

Curran, P. J., Obeidat, K., & Losardo, D. (2010). Twelve Frequently Asked Questions About Growth Curve Modeling. *Journal of Cognition and Development : Official Journal of the Cognitive Development Society*, *11*(2), 121–136. https://doi.org/10.1080/15248371003699969

Piray, P., Dezfouli, A., Heskes, T., Frank, M. J., & Daw, N. D. (2019). Hierarchical Bayesian inference for concurrent model fitting and comparison for group studies. *PLOS Computational Biology*, *15*(6), e1007043. https://doi.org/10.1371/journal.pcbi.1007043

Rigoux, L., Stephan, K. E., Friston, K. J., & Daunizeau, J. (2014). Bayesian model selection for group studies—Revisited. *NeuroImage*, *84*, 971–985. https://doi.org/10.1016/j.neuroimage.2013.08.065

Singer, J. D., & Willett, J. B. (2003). *Applied longitudinal data analysis: Modeling change and event occurrence*. Oxford University Press.

*Figure S1.* Interactions between block, task version, completion of both tasks, and lifetime childhood stress. A) There was an interaction between task version and block for points won (*β* = 2.47, *SE* = 0.96, *p* = .01), such that children who completed both task versions won more points in the volatile task (second visit). B) There was an interaction between lifetime stress exposure, task version, and block for likelihood of choosing high probability of reward choice (*β* = .02, *SE* = .01, *p* = .04) such that there was a negative relationship between lifetime stress exposure and choosing the high probability option only for the stable block in the volatile task version (*β* = -.02, *SE* = .01, *p* = .02). C) There was an interaction between lifetimes stress exposure and task version on the stability regressor (*β* = -.06, *SE* = 0.03, *p* = .04) such that lifetime stress exposure was positively with staying with the prior choice following stable trials in the somewhat volatile task version (*β* = 0.03, *SE* = 0.02, *p* = .18) and negatively in the volatile task version (*β* = -0.03, *SE* = 0.02, *p* = .15). D) There was an interaction between lifetime stress exposure, block, and task version on the volatility parameter (*β* = -.01, *SE* = .005, *p* = .02), such that the effects of lifetime stress on the volatility parameter were most pronounced at increased levels of volatility but only in the somewhat volatile task version (*β* = .02, *SE* = .01, *p* = .003).


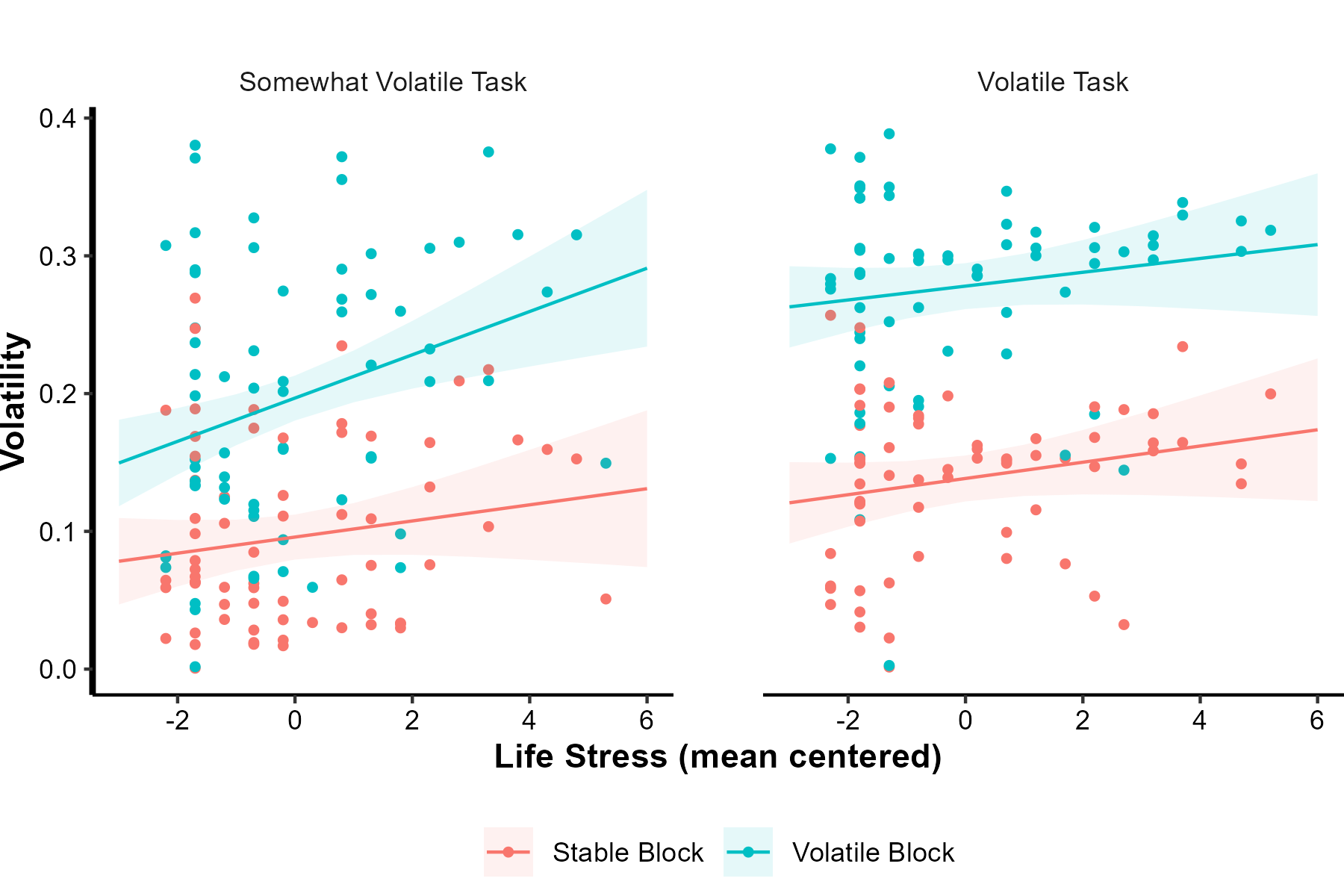


D)


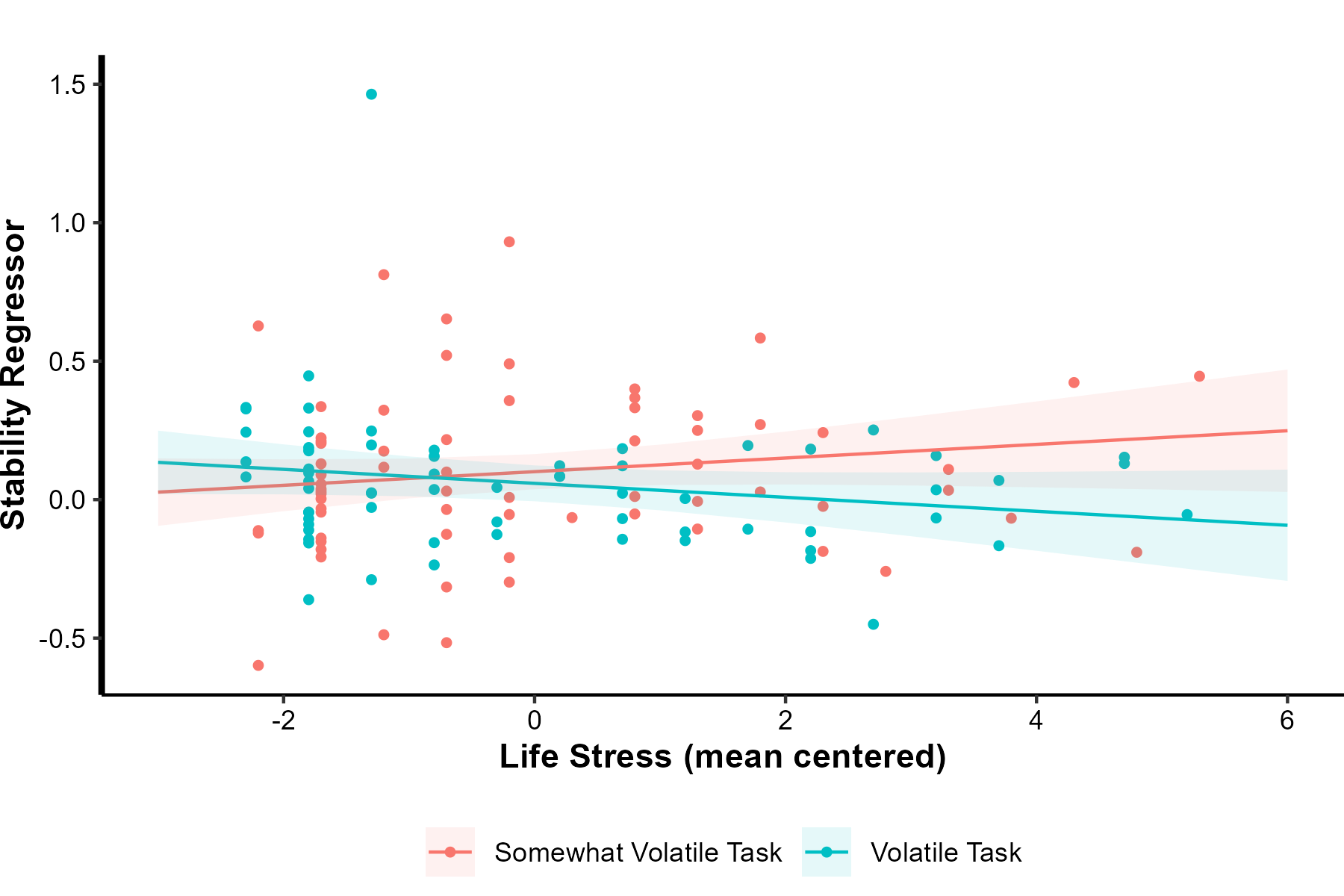


C)


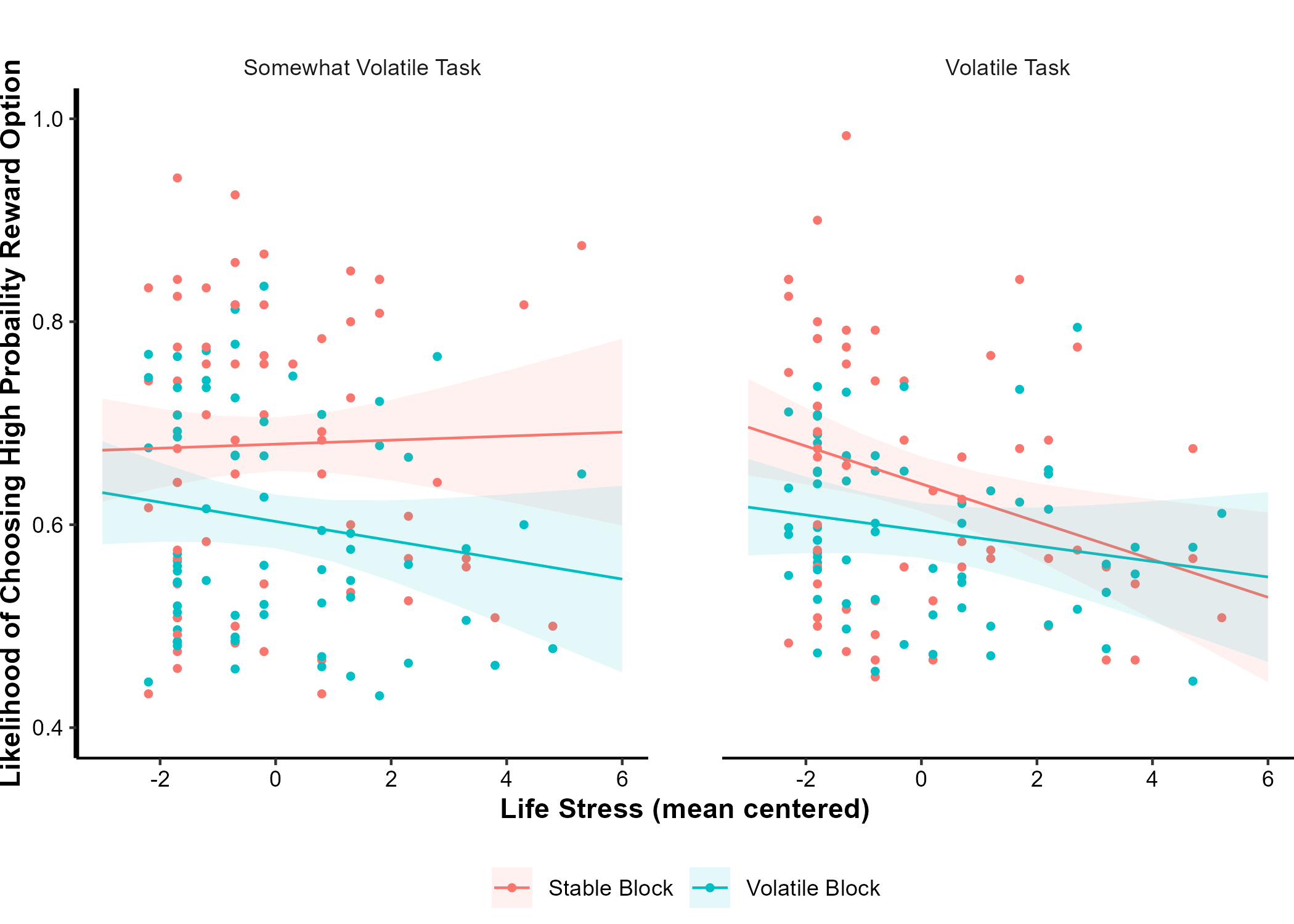


B)


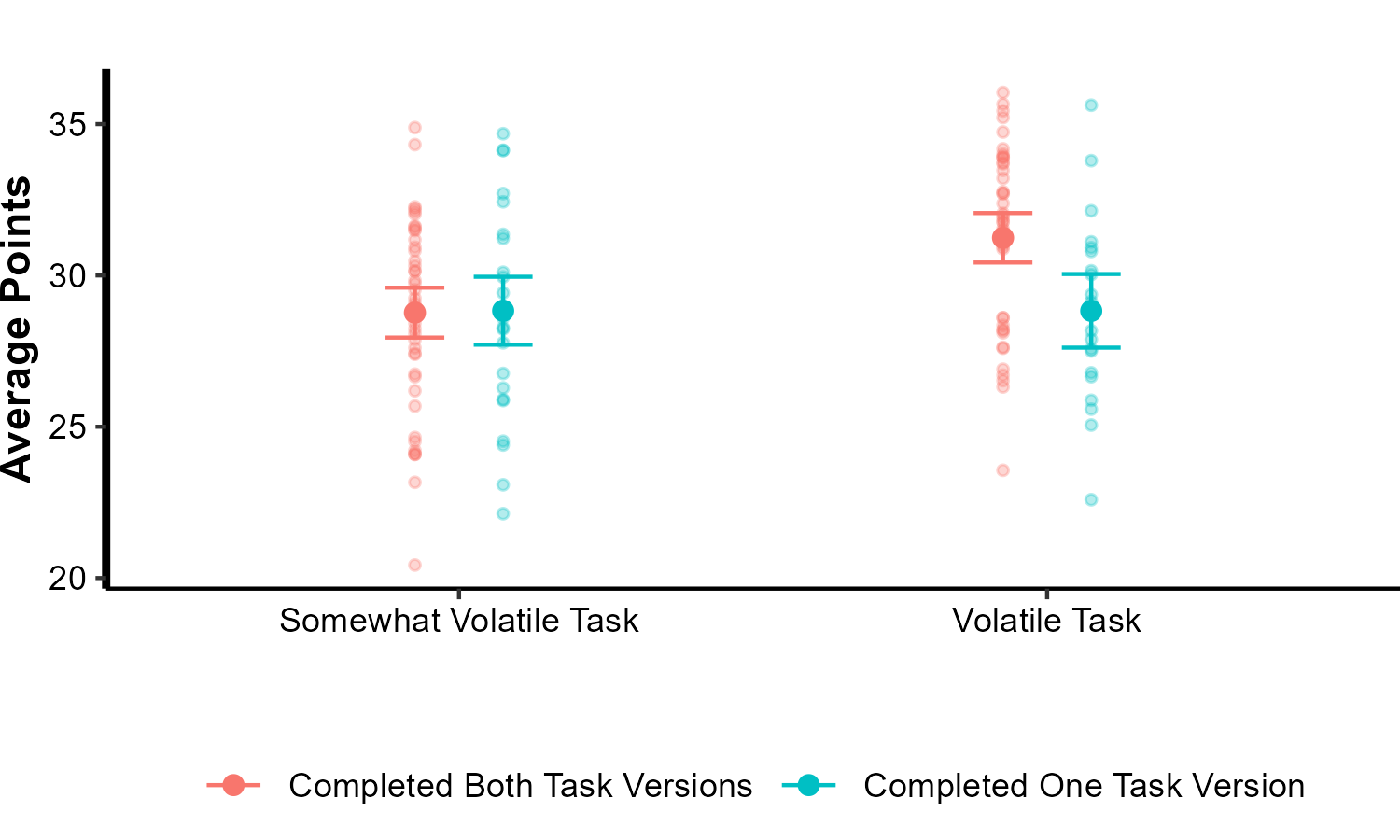


A)

**e**

C)

B)

A)

*Figure S2.* Effects of childhood life stress on theory neutral parameters. A) High lifetime childhood stress was associated with less likelihood of staying with the prior choice (e.g. reduced intercept indicative of increased switching; *β* = -0.05, *SE* = 0.03, *p* = .05). B) High lifetime childhood stress was associated with reduced sensitivity to prior reward (e.g. reward regressor; *β* = -0.001, *SE* = 0.0004, *p* = .04), although this effect was no longer significant after controlling for all covariates. C) There was no effect of lifetime childhood stress on the stability regressor (*β* = -0.004, *SE* = 0.01, *p* = .72).


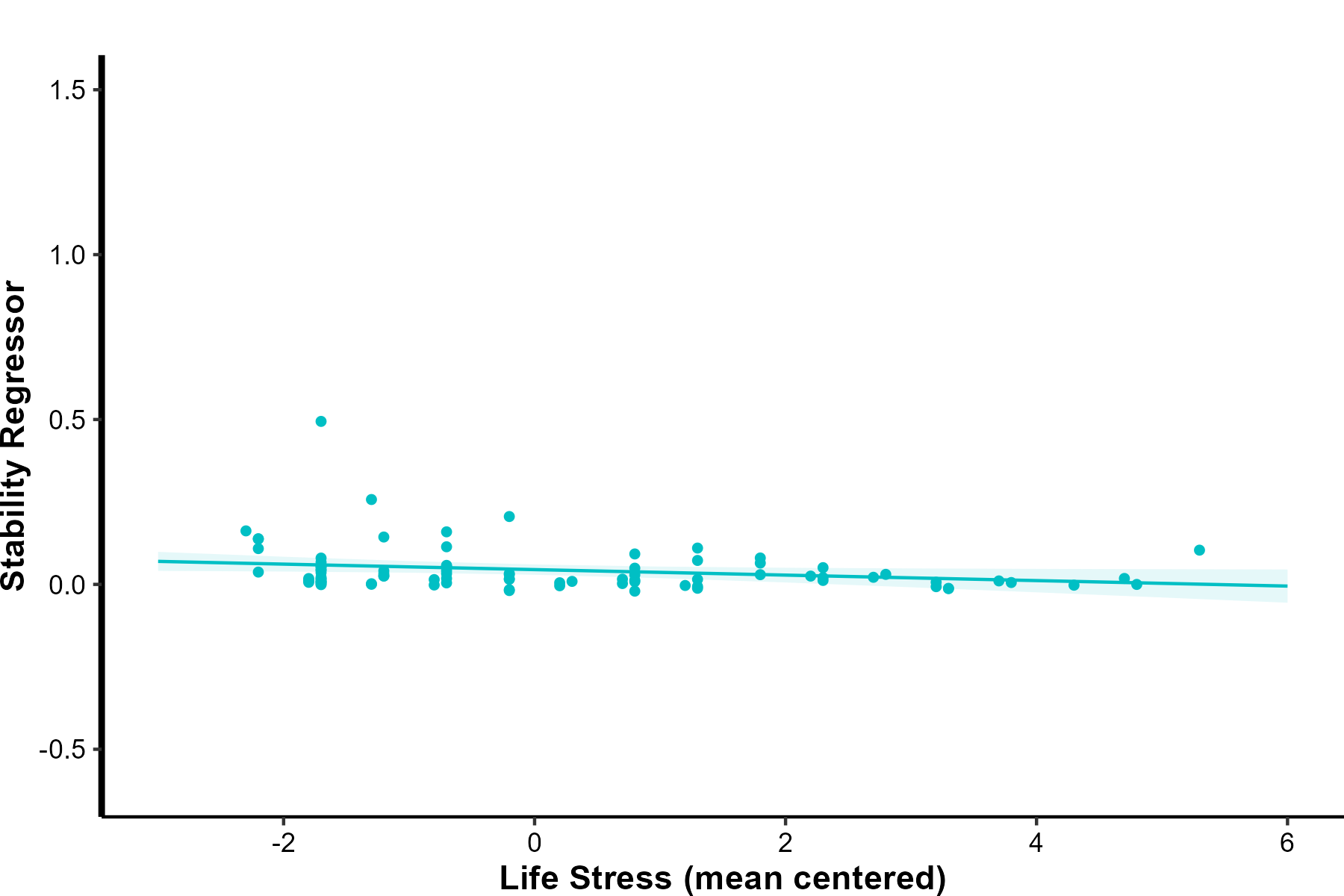


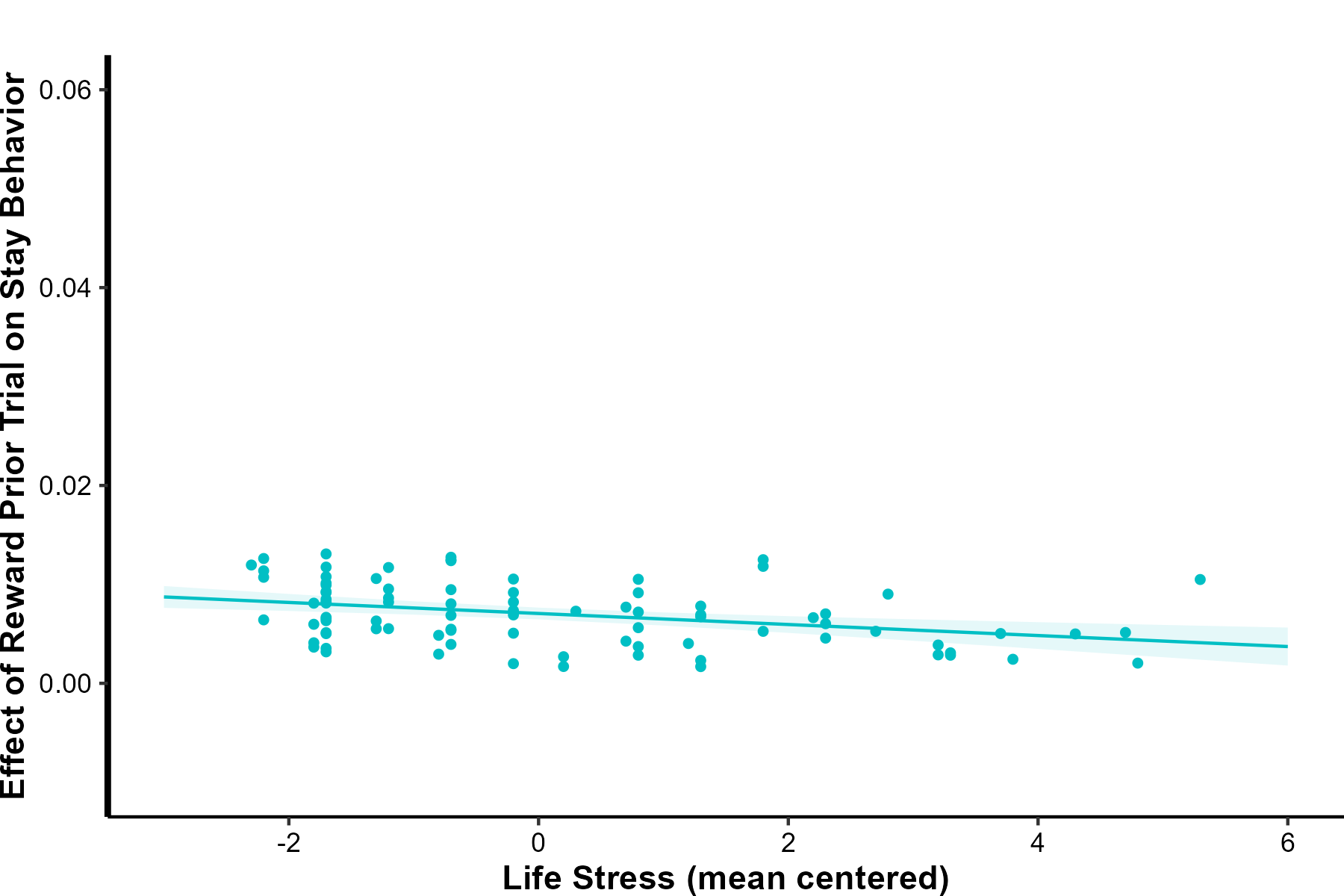


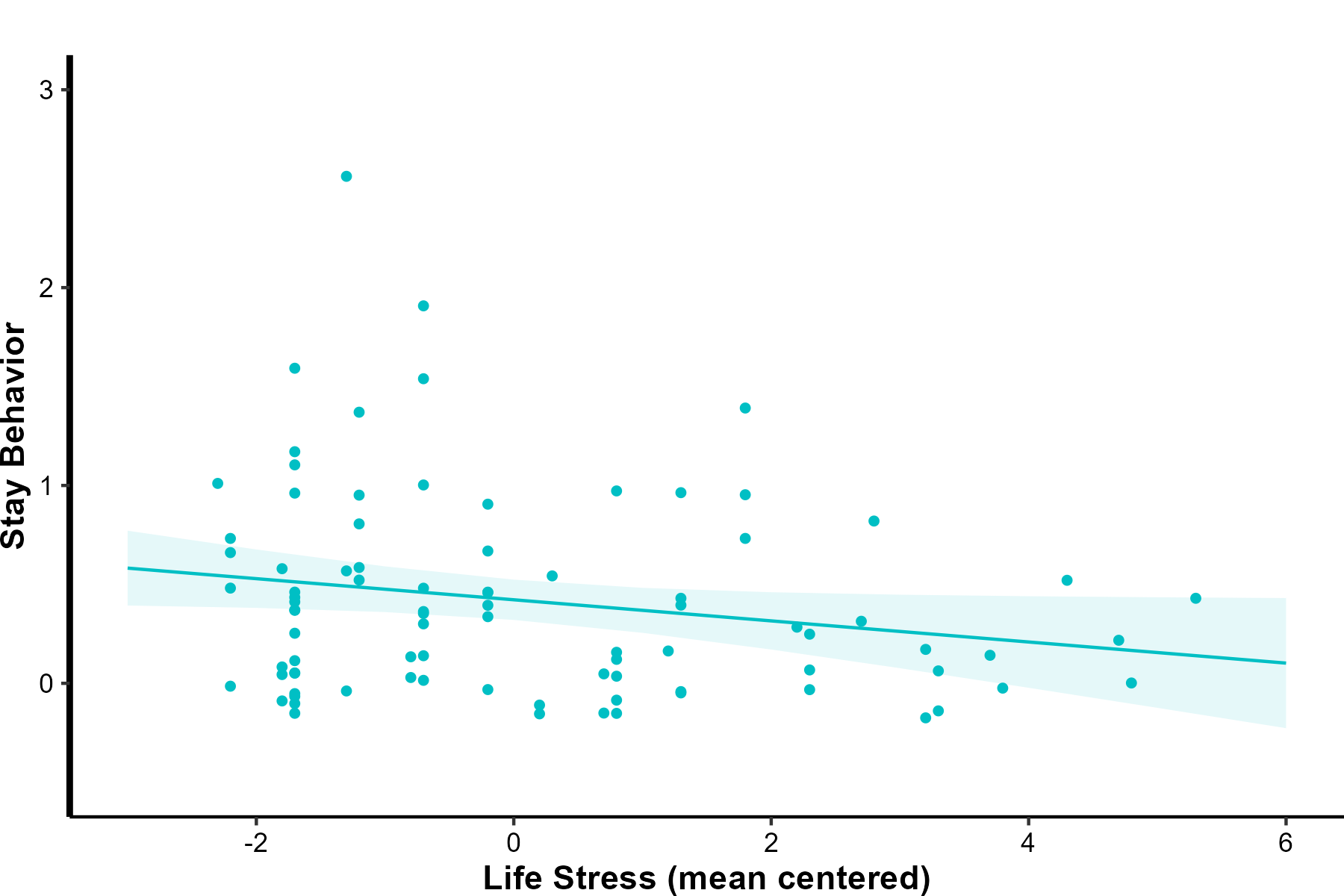


B)


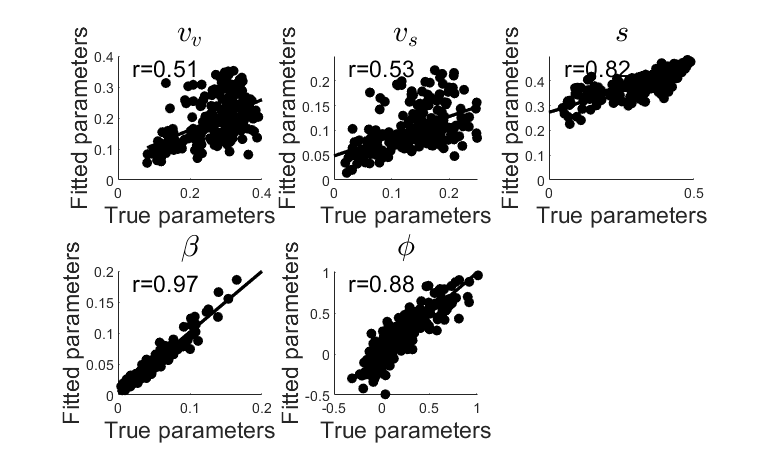


A)


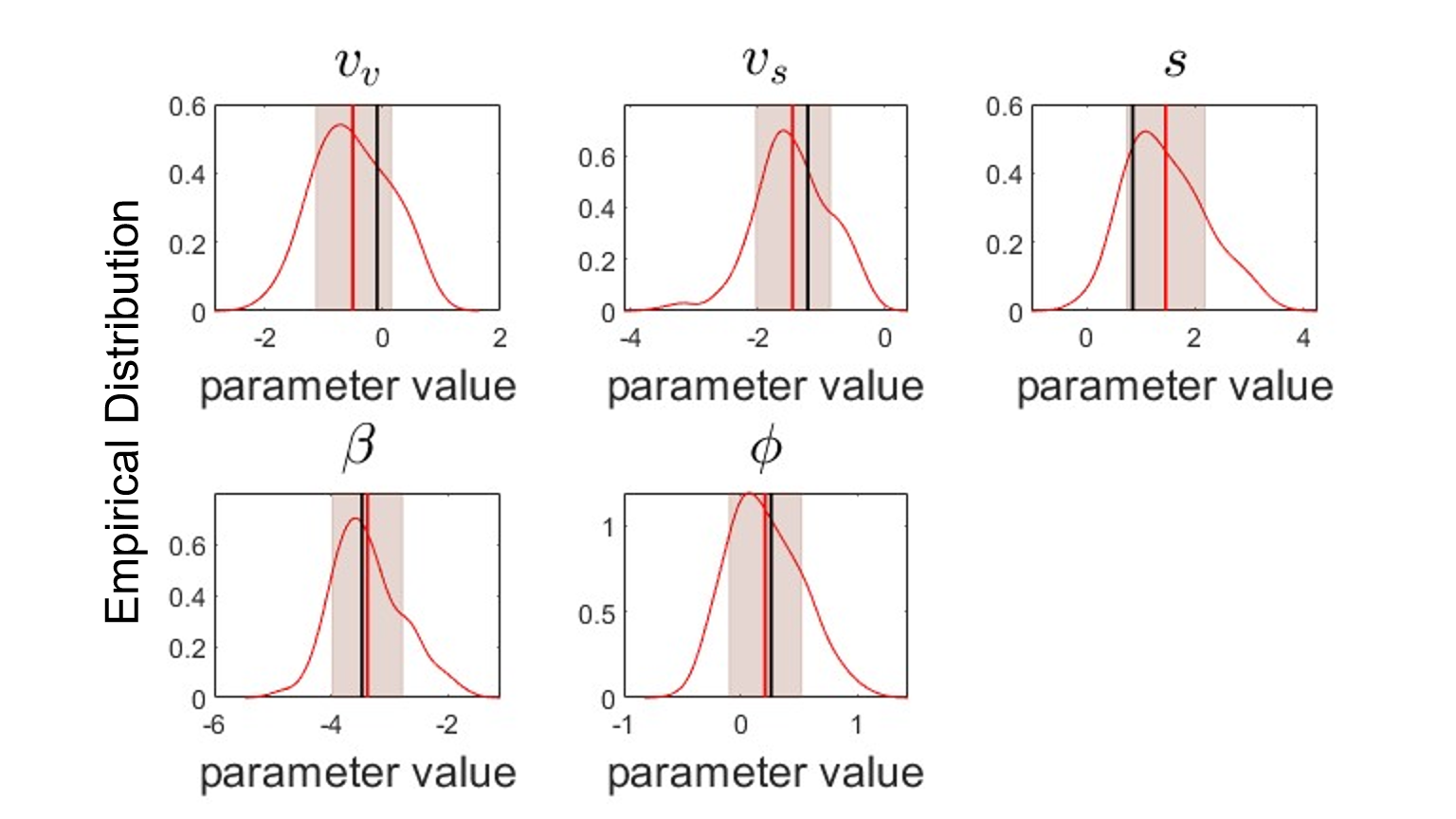


*Figure S3.* Recovery analyses of model parameters. We created synthetic datasets by simulating choice of the model in the full range of fitted parameters. The synthetic dataset was the subject to the same fitting procedure used for actual data. A) Empirical distribution of the recovered parameters is plotted, with the shaded area indicating one standard deviation from the mean (red line). The black line indicates the mean of the true parameters. B) Scatter-plots of true and fitted (recovered) parameters are shown. The r-value indicates Pearson correlation.

C)

B)

A)


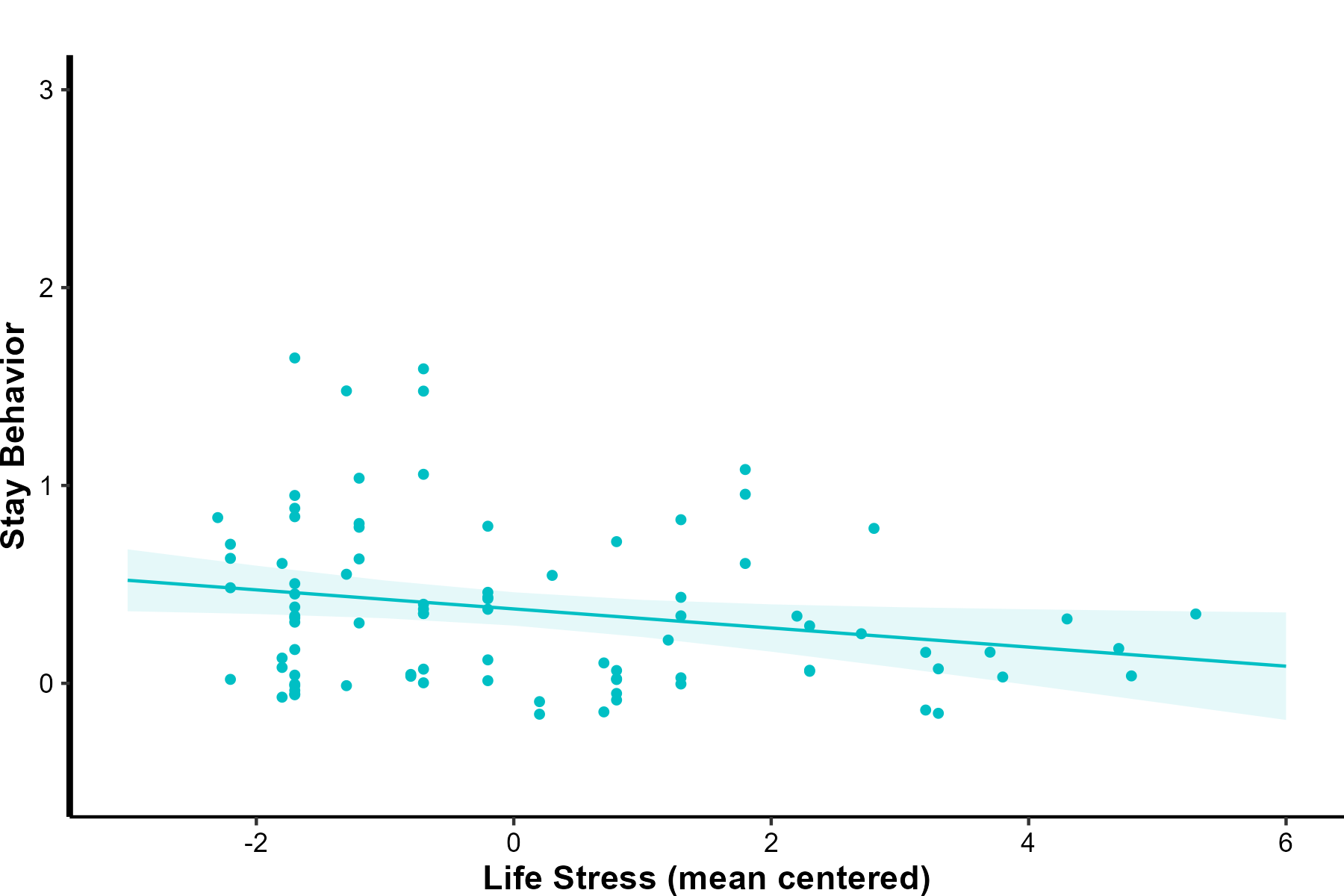


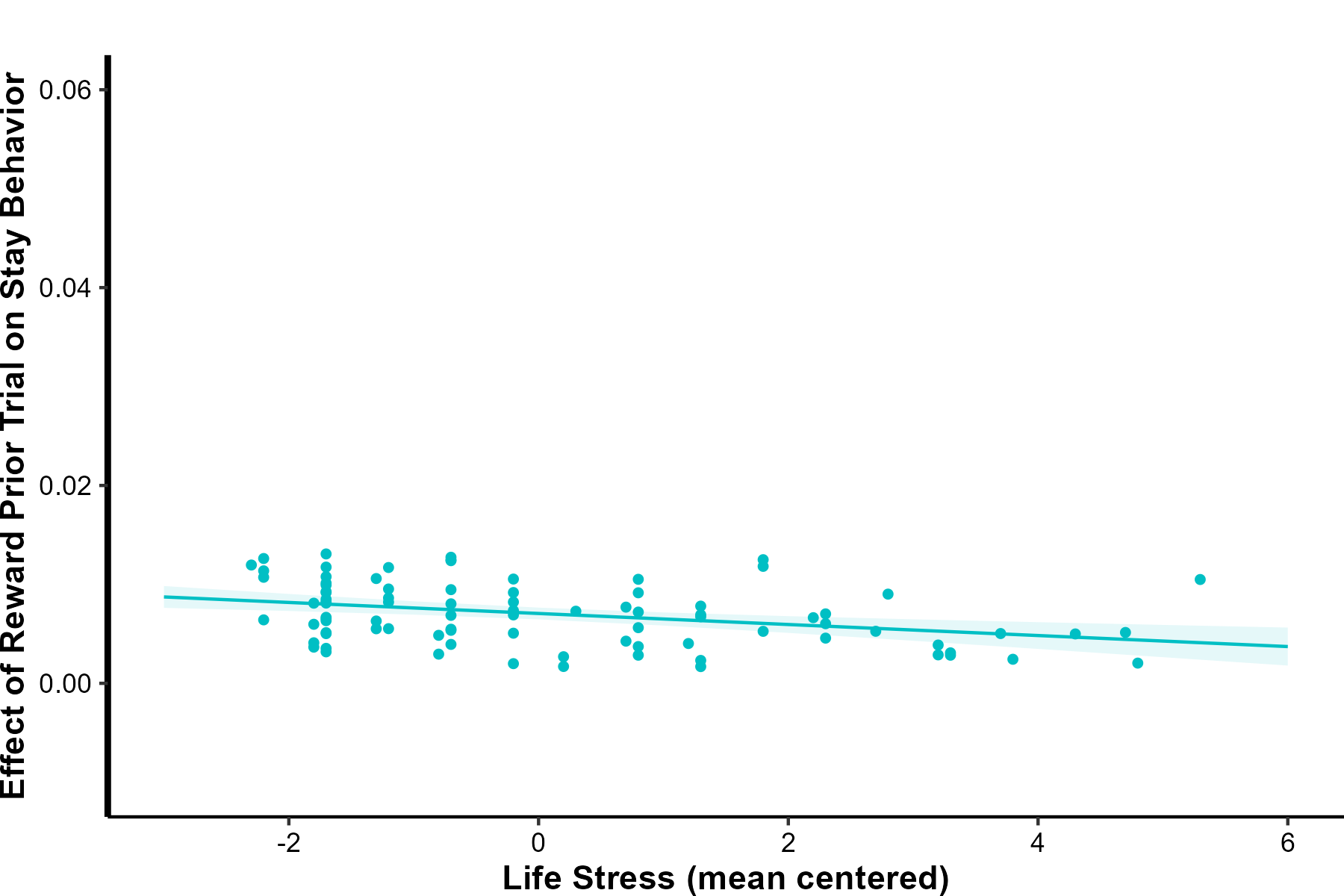


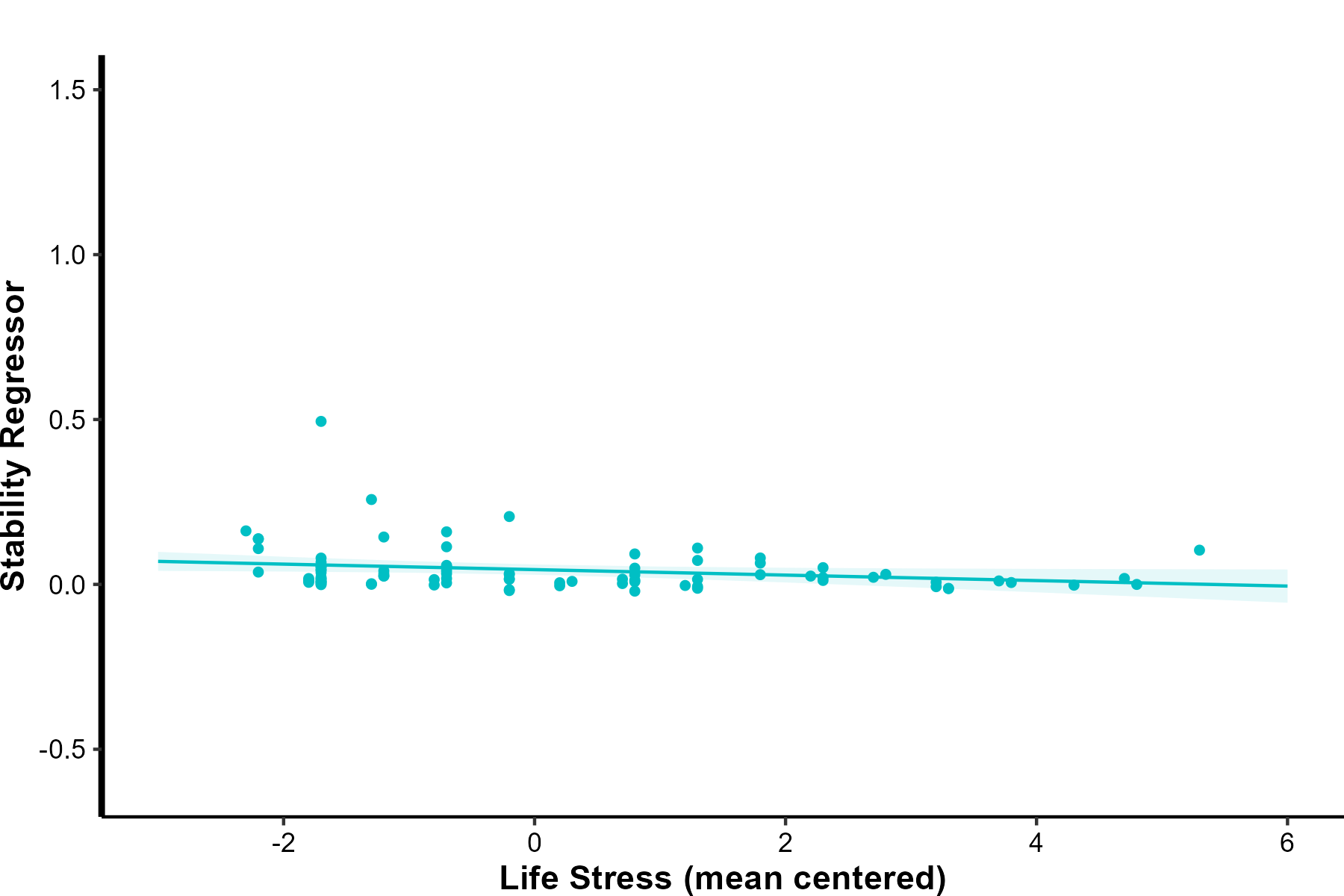


*Figure S4.* Posterior predictive checks found similar patterns of lifetime childhood stress on estimated theory neutral parameters. A) Stay behaviors (intercept: *β* = -0.05, *SE* = 0.02, *p* = .03). B) Reward regressor (*β* = -0.001, *SE* = 0.0002, *p* < .001). C) Stability regressor (*β* = -0.01, *SE* = 0.004, *p* = .04).

\


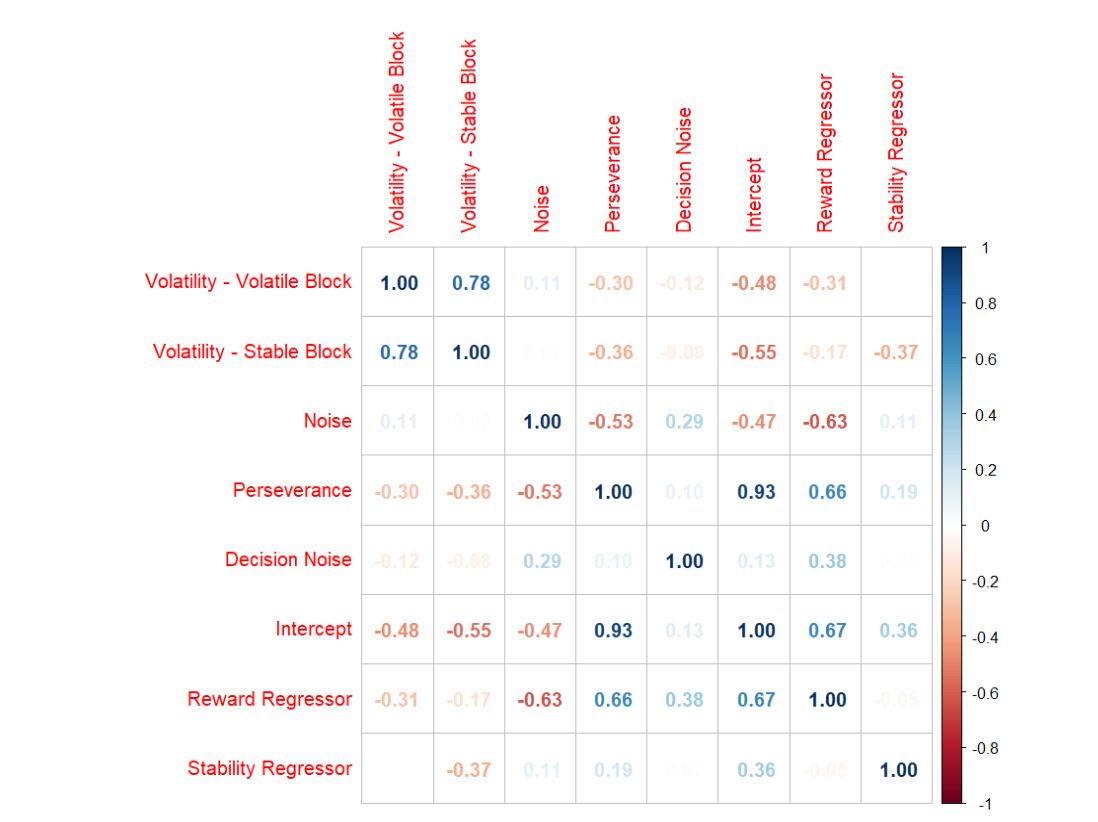


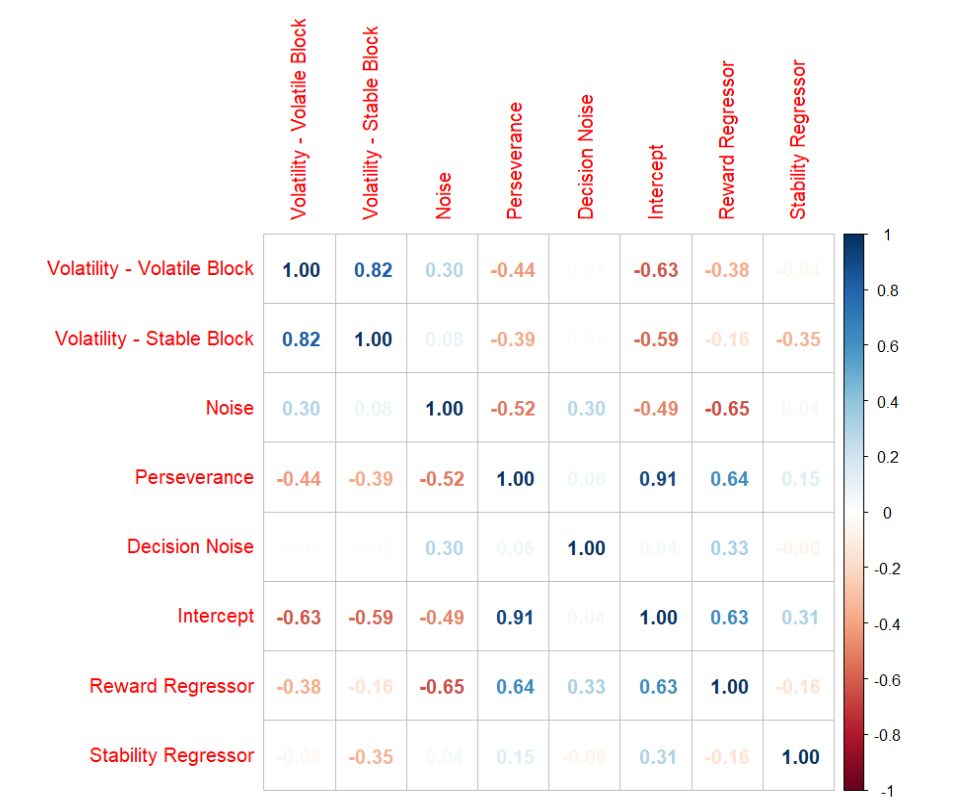


*Figure S5.* Correlations between model parameters and theory neutral (model free) parameters.

B)

A)

*Figure S6.* Effects of lifetime stress exposure on likelihood of choosing high probability reward option (A) and proportion of choices rewarded.


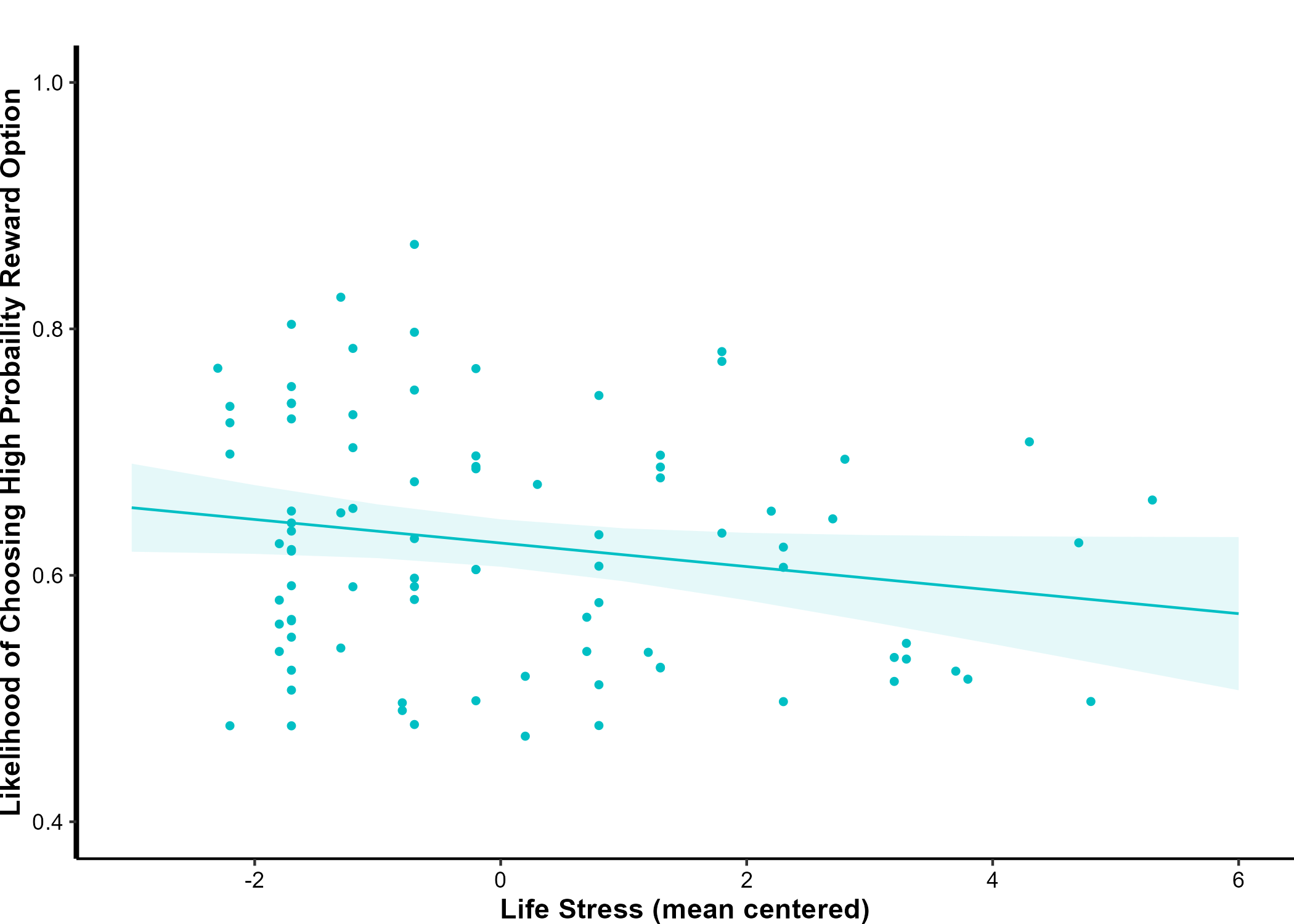


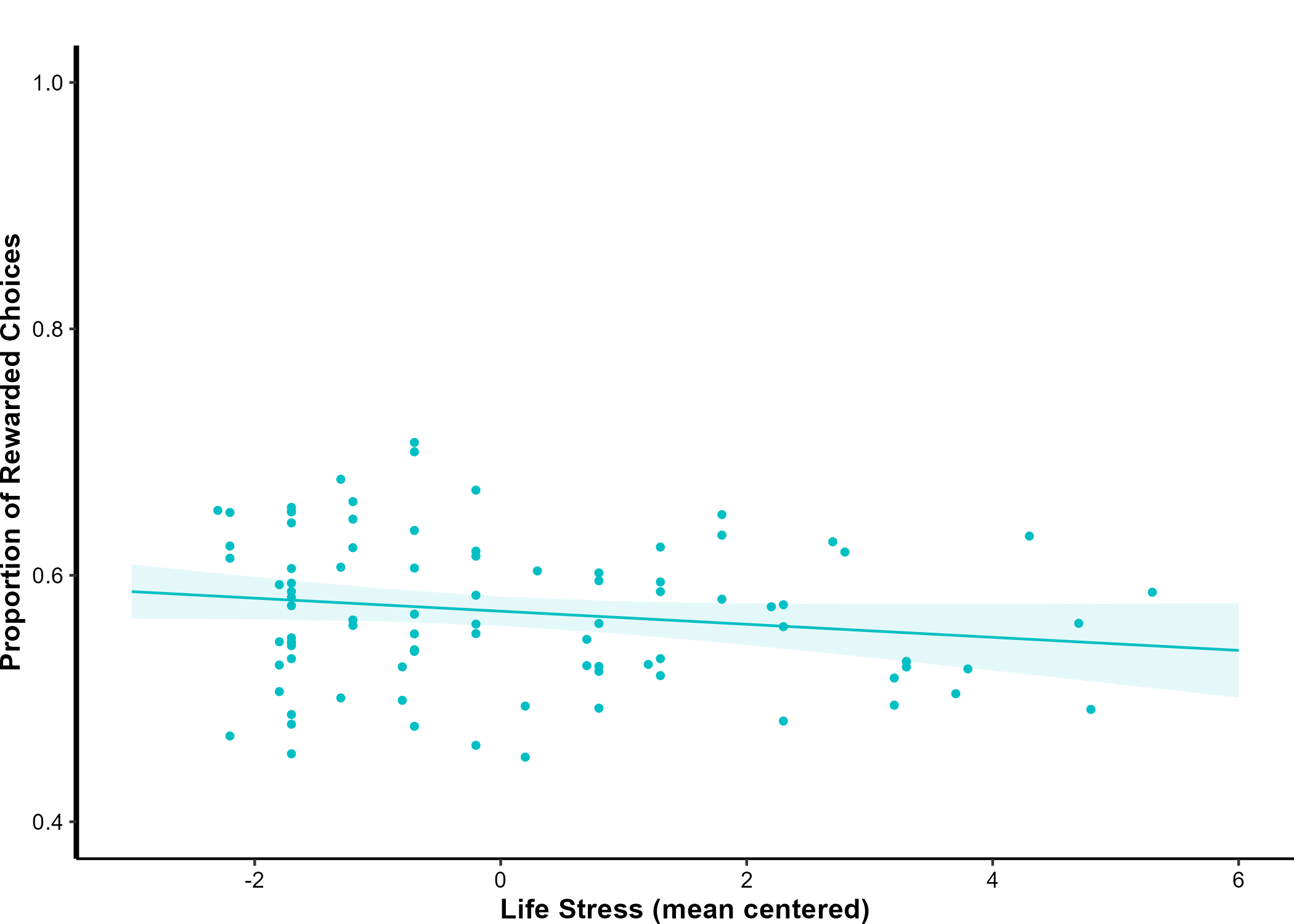


*Figure S7.* Effects of lifetime stress exposure on perseverance (A) and decision coefficient parameters (B).


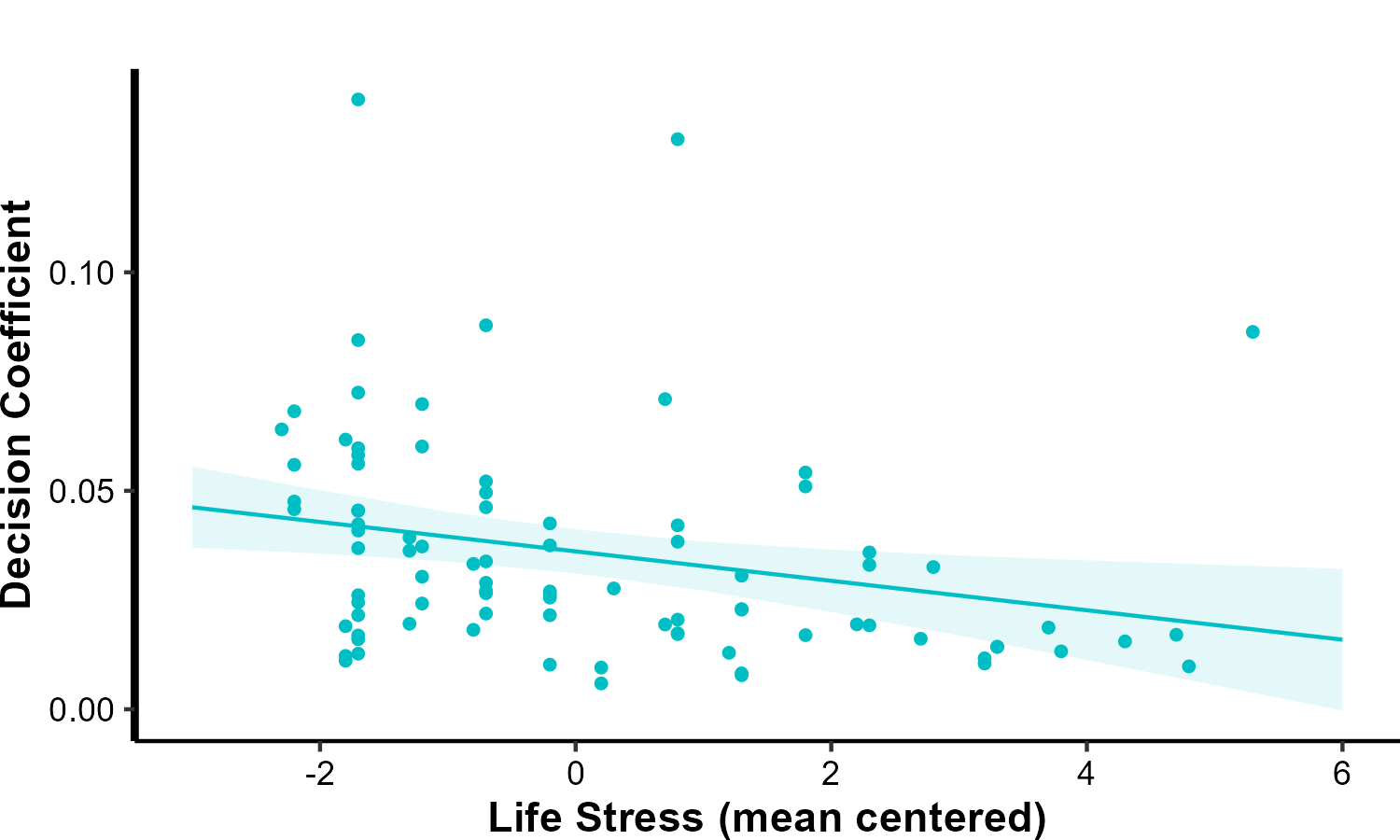


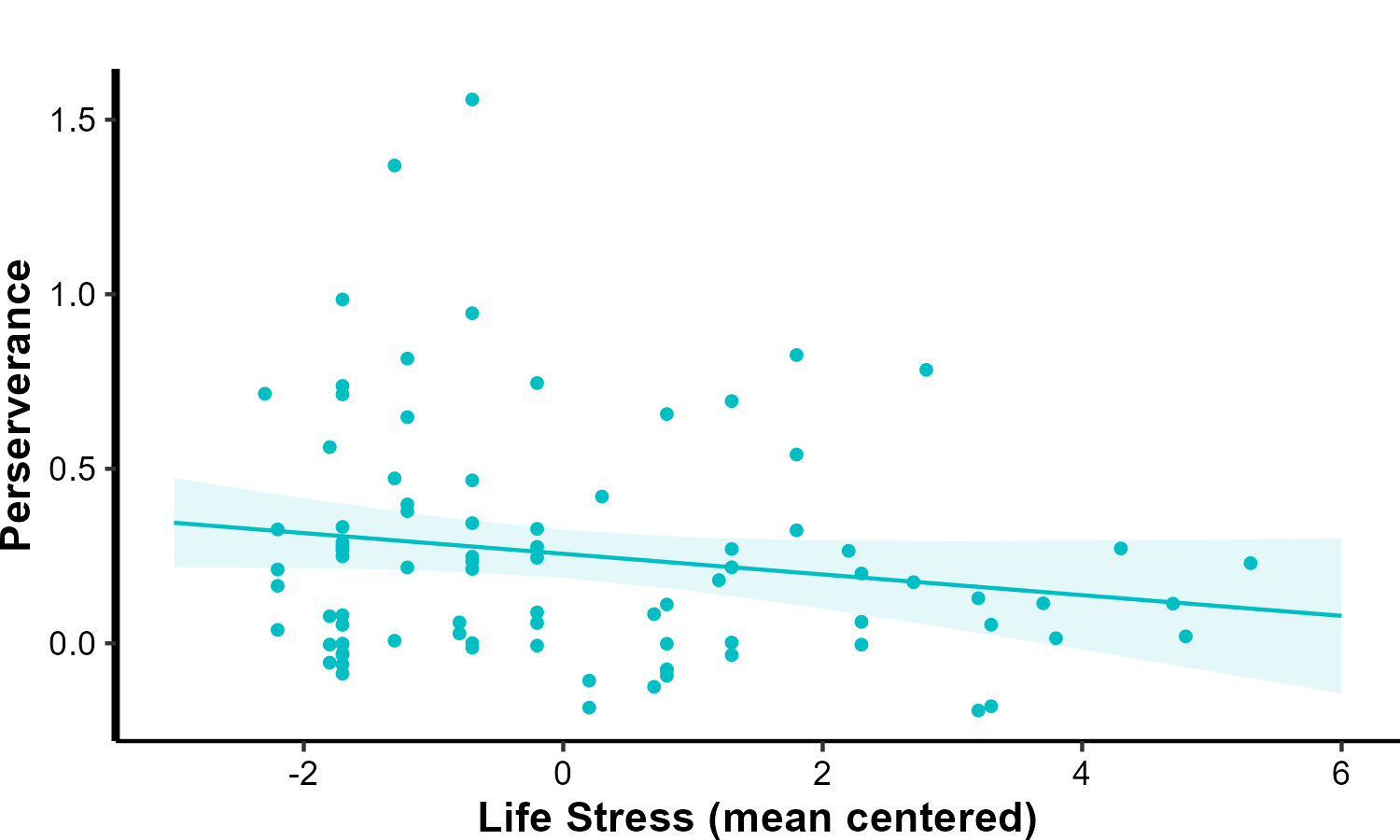


B)

A)
